# Copy Number of the Transposon, *Pokey*, in rDNA Is Positively Correlated with rDNA Copy Number in *Daphnia obtusa*

**DOI:** 10.1371/journal.pone.0114773

**Published:** 2014-12-09

**Authors:** Kaitlynn LeRiche, Shannon H. C. Eagle, Teresa J. Crease

**Affiliations:** Department of Integrative Biology, University of Guelph, Guelph, Ontario, N1G 2W1, Canada; Michigan State University, United States of America

## Abstract

*Pokey* is a class II DNA transposon that inserts into 28S ribosomal RNA (rRNA) genes and other genomic regions of species in the subgenus, *Daphnia*. Two divergent lineages, *Pokey*A and *Pokey*B have been identified. Recombination between misaligned rRNA genes changes their number and the number of *Pokey* elements. We used quantitative PCR (qPCR) to estimate rRNA gene and *Pokey* number in isolates from natural populations of *Daphnia obtusa*, and in clonally-propagated mutation accumulation lines (MAL) initiated from a single *D. obtusa* female. The change in direction and magnitude of *Pokey* and rRNA gene number did not show a consistent pattern across ∼87 generations in the MAL; however, *Pokey* and rRNA gene number changed in concert. *Pokey*A and 28S gene number were positively correlated in the isolates from both natural populations and the MAL. *Pokey*B number was much lower than *Pokey*A in both MAL and natural population isolates, and showed no correlation with 28S gene number. Preliminary analysis did not detect *Pokey*B outside rDNA in any isolates and detected only 0 to 4 copies of *Pokey*A outside rDNA indicating that *Pokey* may be primarily an rDNA element in *D. obtusa*. The recombination rate in this species is high and the average size of the rDNA locus is about twice as large as that in other *Daphnia* species such as *D. pulicaria* and *D. pulex*, which may have facilitated expansion of *Pokey*A to much higher numbers in *D. obtusa* rDNA than these other species.

## Introduction

Transposable elements (TEs) are mobile segments of DNA that can excise from one genomic location and insert into another, and they comprise a large portion of the genomes of many organisms [1]. TEs exhibit broad diversity in both structure and mechanisms of transposition, and host genomes vary widely with respect to the number of TEs they contain, which partly accounts for the large differences in genome size among related species [2], [3]. Active TEs are usually considered to be detrimental to their host because they can affect genome structure and function. For example, chromosome breakage, ectopic recombination and genome rearrangement are some common, potentially deleterious consequences of TE insertions [4]. TEs also act as mutagens when they alter gene expression after inserting into regulatory sequences, or gene structure after inserting into exons (reviewed in [5]). TEs may also have indirect effects on nearby genes that are mediated by the epigenetic mechanisms typically used by the host to silence them (reviewed in [3]). TE persistence in the genome is a balance between mechanisms that decrease copy number (such as negative selection by the host or mutational degradation) and those that increase it (such as transposition by the TE or recombination and DNA repair by the host) [3], [4]. On the other hand, some TEs have also been co-opted by their host for useful functions such as telomeres in *Drosophila* (reviewed in [6]) and beneficial structural rearrangements of host DNA such as those associated with V(D)J recombination in jawed vertebrates [7]. Even so, such beneficial effects appear to be much less common than detrimental effects caused by TEs.

Ribosomal DNA (rDNA) in eukaryotes is a multigene family of repeat units containing genes encoding 18S, 5.8S, and 28S rRNA (hereafter referred to as 18S, 5.8S or 28S genes), which form the catalytic core of ribosomes. The rDNA repeats are typically organized in tandem arrays on one or more chromosomes, and their copy number varies from tens to tens of thousands per haploid genome [8]. rRNA genes are the most transcribed and conserved eukaryotic genes because large quantities of ribosomes are needed to translate mRNA, especially during periods of growth [9]. rDNA usually evolves in a concerted fashion, such that repeat units within species remain highly homogeneous despite their divergence between species [9]. Sequence homogeneity among repeat units depends on the size of the rDNA locus and on relative rates of mutation and recombination, including unequal gene conversion and crossing over between misaligned copies [10], both of which change the number of rDNA units in an array.

Given the intensity of purifying selection on and the concerted evolution of rRNA genes, it is surprising that some TEs insert into specific locations in rDNA [9]. On the other hand, it has been argued that rDNA may be an ideal niche for TEs because it is very repetitive, and increases in rRNA gene number due to recombination could generate new insertion sites. In addition, rRNA genes are highly transcribed enabling regular expression of TE-encoded genes ([11], [12] and references within).

TE insertions in 18S or 28S genes often render the genes non-functional [13] suggesting that selection should operate against the TEs. However, because organisms generally have many more rDNA units than they require for viability, TE insertions below a particular threshold number may have little effect on host fitness [11]. Indeed, some rDNA-specific TEs have persisted within eukaryotic lineages for hundreds of millions of years. For example, R1 and R2 are Class I non-LTR retrotransposons that insert into specific sites in 28S genes and are present in most lineages of arthropods that have been tested [11]. R2 also occurs in many other animal phyla and may predate the divergence of protostomes and deuterostomes [13]. The wide distribution of these TEs has been attributed to vertical transmission in rDNA and divergence in parallel with the host [13].

*Pokey* is a Class II DNA TE that was originally found in the cladoceran crustacean, *Daphnia pulex* [14] but has since been identified in many species in the subgenus *Daphnia* [15]. Closely related TEs have also been identified in the silkmoth, *Bombyx mori* [16]; the tunicate, *Ciona savignyi* [17]; and the rotifer, *Adineta vaga* [18]. *Pokey* has been classified as a member of the cut-and-paste *piggyBac* superfamily due to its terminal inverted repeats (TIRs), its preference for TTAA insertion sites and the amino acid sequence of the protein encoded by its putative transposase gene [15], [19]. The putative transposase has been shown to excise non-autonomous *Pokey* elements in a yeast excision assay suggesting that *Pokey* is indeed an active TE [20].

Two lineages of *Pokey* have been identified in *Daphnia*; *Pokey*A, which was originally found in *D. pulex* [14] but is widespread in the subgenus *Daphnia* [21], and *Pokey*B, which was originally found in *Daphnia obtusa* [21] but also occurs in the *D. pulex* species complex [22]. Phylogenetic analysis shows that *Pokey* has undergone stable, vertical inheritance in *Daphnia* 28S genes for tens of millions of years [21]. The 28S TTAA site into which it inserts is only 4 nt away from the R2 insertion site and 64 nt away from the R1 insertion site. However, unlike R-elements, which specifically target rDNA, *Pokey* also inserts into many TTAA sites outside of rDNA [14], [23], [24].

*Daphnia* typically reproduce via cyclic parthenogenesis, which involves alternation between production of offspring via apomictic (clonal) parthenogenesis and production of haploid eggs that require fertilization. McTaggart et al. [25] used clonally-propagated mutation accumulation lines (MAL) of *D. obtusa* descended from a single “wild-caught” female to estimate the rate of recombination in rDNA, which occurs as a single array of repeat units in *D. pulex* [26]. They measured the change in relative frequency of length variants in the 18S gene and found the recombination rate to be fairly high at 0.02 to 0.06 events per generation. They also observed changes in the number of 18S genes (both increases and decreases) of up to 80% across 90 generations (as high as 5.5% per generation).

Because a single individual is used to propagate each new generation of the MAL, natural selection is relaxed allowing the accumulation of deleterious mutations, including TEs, and unusually large or small rDNA arrays. In this study, we measure the number of *Pokey* elements as well as 18S and 28S genes after ∼87 generations in these MAL. We expected changes in 18S and 28S gene number to be similar as they both occur in each rDNA unit. We also expected changes in *Pokey* number to be strongly correlated with changes in 28S number if *Pokey* elements are dispersed throughout rDNA arrays. Conversely, if *Pokey* elements tend to be clustered in the rDNA locus, and recombination events are biased to exclude units whose 28S gene contains an insert, then changes in *Pokey* number may not be strongly correlated with overall changes in 28S number [12]. To compare the level and range of copy number variation generated during clonal reproduction in the laboratory MAL, we also surveyed *Pokey* and rRNA gene number in 21 *D. obtusa* isolates from 15 natural populations, whose rRNA gene and *Pokey* numbers are expected to be under selective constraint.

## Methods

### Mutation accumulation lines of D. obtusa

Fifty mutation accumulation lines (MAL) were initiated from the clonally-produced daughters of a single *D. obtusa* female collected from a pond in Trelease Woods near Urbana, Illinois in 2001 [25]. Environmental conditions were controlled in the laboratory to promote clonal reproduction and each generation, a single daughter was randomly chosen to represent the next generation. The goal was to minimize the operation of natural selection within each line to allow accumulation of deleterious mutations by genetic drift. Details on the propagation of the MALs can be found in McTaggart et al. [25].

The current study used the four “fine-grained” lines (MAL-FG: MAL3, 12, 29, 30) that were each sampled approximately every five generations until generation 95 [25], and an additional 16 MAL sampled once between generation 80 and 90 (mean  =  generation 87, S1 Table). Including the last isolate of each MAL-FG gives a total of 20 isolates sampled at ∼87 generations, and they are referred to as the MAL-87 isolates. All MAL isolates are coded according to their line and generation number (i.e. 02-89 is line 2 sampled at generation 89).

The MAL were sampled by allowing the sisters of the focal individual (the backups) to clonally produce a brood of offspring (5 to 20 individuals) and then pooling these individuals to create an “isolate” for subsequent genetic analyses. Together, the *Pokey* and rDNA gene number of these individuals should be representative of the single female that gave rise to this generation. DNA was extracted from the MAL isolates by McTaggart et al. [25] using the CTAB method [27]. A NanoDrop ND-8000 spectrophotometer (Wilmington, Delaware, USA) was used to determine the concentration (ng/µL) of each DNA sample. We used Transposable Element Display (TED) to estimate *Pokey*A number outside rDNA (g*Pokey*) at five time points between generation 5 and 85 from each of the four MAL-FG (20 isolates). We used quantitative PCR (qPCR) to estimate rRNA gene number and the number of *Pokey*A and *Pokey*B inserted in 28S genes (r*Pokey*) at seven times points between generation 5 and 85 from each of the MAL-FG (28 isolates), and in the 16 additional MAL-87 isolates.

### Natural populations

**Figure 1:**
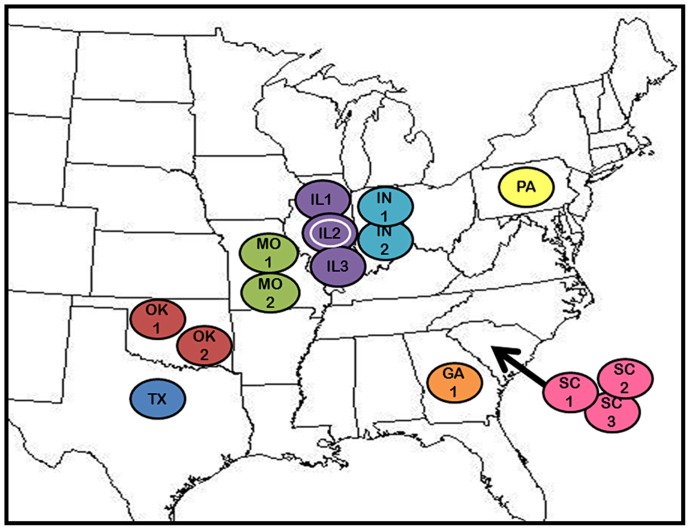
Natural populations of *Daphnia obtusa* sampled for this study. Twenty-one isolates were obtained from 15 populations in the eastern and midwestern USA. Codes indicate the state and population number. The white circle indicates the population from which the progenitor female of the mutation accumulation lines was sampled.

Twenty-one isolates were sampled with a plankton net from 15 natural populations (NP) in the eastern United States, including ponds in Georgia, Illinois, Indiana, Missouri, Oklahoma, Pennsylvania, South Carolina and Texas (Fig. 1, S1 Table in S1 File). *Daphnia* were sampled from ponds accessed via public roadsides or on private land with the permission of the land owner. No specific permissions are required to sample *Daphnia* as they are not endangered or protected species.

Cultures were initiated in the laboratory from single females that were allowed to propagate clonally to increase the tissue available for DNA extraction [27]. DNA was extracted using the GenElute genomic DNA extraction kit (Sigma Chemical, St. Louis, MO, USA). A NanoDrop ND-8000 spectrophotometer was used to determine the concentration (ng/µL) of each sample. Isolates were coded based on their state, population and individual number. For example, IL1.2 is individual 2 from population 1 in Illinois. All 21 isolates, hereafter referred to as the NP isolates, were analyzed using qPCR but only 19 of them were analyzed using TED due to lack of sufficient DNA from the other two samples.

### Transposable Element Display

Transposable Element Display (TED) tends to underestimate TE copy number [28] but the only way to estimate *Pokey* number in other genomic locations (g*Pokey*) using qPCR is to estimate total *Pokey* number and then subtract r*Pokey* from it [28]. Our preliminary results indicated that g*Pokey*A number was very low but r*Pokey*A number was high in our *D. obtusa* isolates. Given the experimental variation in qPCR results (discussed below), we decided to use TED to estimate g*Pokey*A number.

TED was performed as described in Valizadeh and Crease [23] except that we used an annealing temperature of 50°C for both the primary and secondary PCRs. Briefly, 100 ng of DNA from each sample were digested with the *Bfa*I restriction enzyme after which the *Bfa*I forward and reverse linkers were ligated to the digested DNA. The ligated DNA was then amplified with a *Pokey*A-specific forward primer and the *Bfa*I reverse primer (S3 Table in S2 File). The primary PCR was done in triplicate. The primary PCR amplicons were used as a template for a secondary PCR that used a fluorescently labelled *Pokey*-specific forward primer, which binds downstream of the primary forward primer (S3 Table in S2 File). Secondary PCR amplicons were diluted 1∶20 and separated by size on an ABI 3730 DNA analyzer in the Genomics Facility at the University of Guelph. The electropherograms were analyzed with Peak Scanner (Applied Biosystems, Forest City, CA, USA). Only amplicon peaks present in at least two of the triplicates and at least 150 units on the fluorescence axis were counted. In addition, only peaks greater than 160 bp long were counted as this is the size of the amplicon that would be generated if a *Bfa*I site occurred immediately downstream of the *Pokey* TTAA insertion site.

### Quantitative PCR

**Figure 2:**
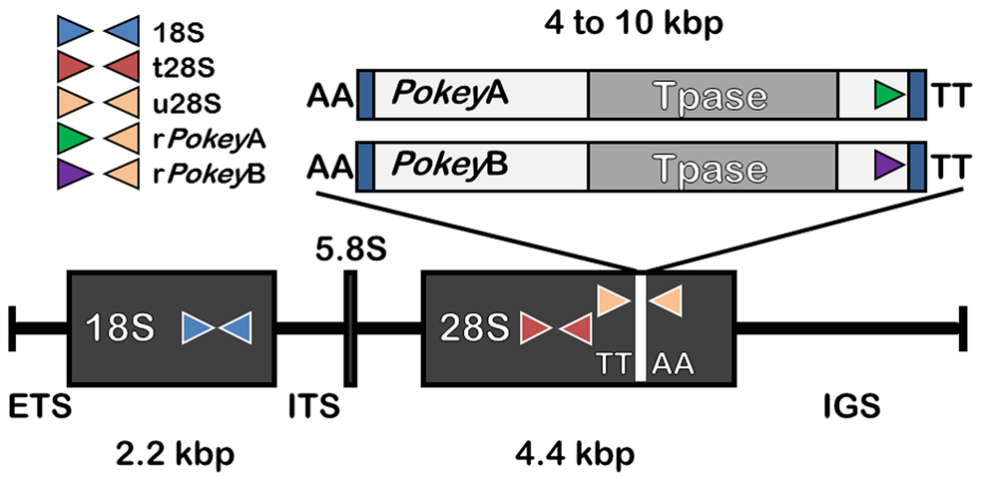
General location of qPCR primers used to estimate rRNA gene and *Pokey* number in *Daphnia obtusa*. Primers are indicated by short arrows. 18S = 18S rRNA coding region, 5.8S = 5.8S rRNA coding region, 28S = 28S rRNA coding region, ETS  =  external transcribed spacer, IGS  =  intergenic spacer. *Pokey* inserts into a specific TTAA site in the 28S gene. Full-length elements have a transposase gene (Tpase) and terminal inverted repeats (vertical blue bars). The length of the 18S gene is based on a sequence from *D. pulex* [36]. The length of the 28S gene is based on a sequence from *D. pulicaria* [37]. The length of complete *Pokey* elements is based on sequences from the *D. pulex* genome [26] and elements cloned from the rDNA of *D. obtusa* (E. Penton and T. Crease, unpublished). The primer sets that amplify *Pokey* in 28S genes (r*Pokey*) are specific to the *Pokey*A (green) or *Pokey*B (purple) lineages. The diagram is not to scale and was redrawn from [28].

qPCR was used to estimate haploid gene numbers by comparing the rate of amplification of a multicopy gene to that of a single-copy gene as in [25] and [28]. The multicopy genes we amplified using qPCR were: r*Pokey*A and r*Pokey*B, total 28S genes (t28S), 28S genes lacking an insert in the *Pokey* TTAA insertion site (u28S), and 18S genes. The single-copy reference genes are *Gtp* (a member of the RAB subfamily of small GTPases) and *Tif* (a transcription initiation factor) (Fig. 2, S4 Table in S2 File). Amplification efficiencies for the primer pairs were estimated by generating standard curves and calculating the Percent Amplification Efficiency (PAE) as described in Eagle and Crease [28]. The PAE was estimated at least three times for each primer pair and any values outside the 95% confidence interval were omitted from calculation of the mean value [22]. qPCR was performed using the 1X PerfeCTa SYBR Green FastMix with ROX (Quanta BioSciences, Gaithersburg, MD, USA) on 10 ng of DNA extracted from isolates of the four MAL-FG sampled at seven time points, 16 MAL-87 isolates, and the 21 NP isolates (S1 Table in S1 File). All reactions were run in triplicate on a StepOnePlus Real-Time PCR System (Applied Biosystems, Foster City, CA, USA). The StepOne software was used to set the baseline, and C_T_ values were obtained from the qPCR amplification plot using the threshold value of 0.2 for 50-bp amplicons. To compensate for the fact that amplicons bind SYBR green dye in proportion to their length, the threshold value for longer amplicons was adjusted according to the formula, threshold = 0.2×(2^∧^[1 - (50/length in bp)] as suggested by [28]. If the standard deviation of the triplicate C_T_ values exceeded 0.2, the most extreme C_T_ value was excluded from the estimation of gene number [28].

Gene number was calculated as 2^−ΔCT^ where ΔC_T_ = [(C_T_ value of the multicopy gene x PAE of that gene) – (C_T_ value of a single-copy reference gene x PAE of that gene)]. The three C_T_ values of each single copy gene (S2 Table in S1 File) were each subtracted from each of the three C_T_ values of each multicopy gene for a total of 18 copy number estimates [(3×3)+(3×3)] if all replicate values were used. The 18 estimates were averaged to give the mean and standard deviation of haploid copy number for each multicopy gene in each sample (S1 Table in S1 File). The haploid numbers were rounded up or down to the nearest 0.5. We also calculated the *Tif:Gtp* ratio (S1 Table in S1 File), which is the number of *Tif* genes relative to *Gtp* genes and is calculated in the same way as multicopy gene number. Multicopy gene estimates were adjusted in isolates with a *Tif:Gtp* ratio of 0.7 (2∶3) or 0.5 (2∶4) (S1 Figure in S3 File), which indicates that there are three or four copies, respectively of the *Gtp* gene instead of the two copies expected [28]. Correlation and regression analyses and t-tests were done in Microsoft Excel. The significance level (0.05) for t-tests comparing 18S and 28S gene number within isolates was adjusted using the sequential Bonferroni technique according to [29].

We estimated the rate of rRNA gene number change per generation by subtracting mean gene number in the four MAL-FG isolates at generation 5 from the number at each end point. We used generation 5 as it is the earliest generation for which we have gene number data. Even though there are only 4 values, the range is much lower (63 for 18S, 20 for 28S) than it is at ∼87 generations (226 for 18S, 237 for 28S). We then divided the difference by the number of generations in each time interval for the MAL-FG isolates, and across the total number of generations for both MAL-FG and MAL-87 isolates.

## Results

### Variation in rRNA gene number

**Figure 3:**
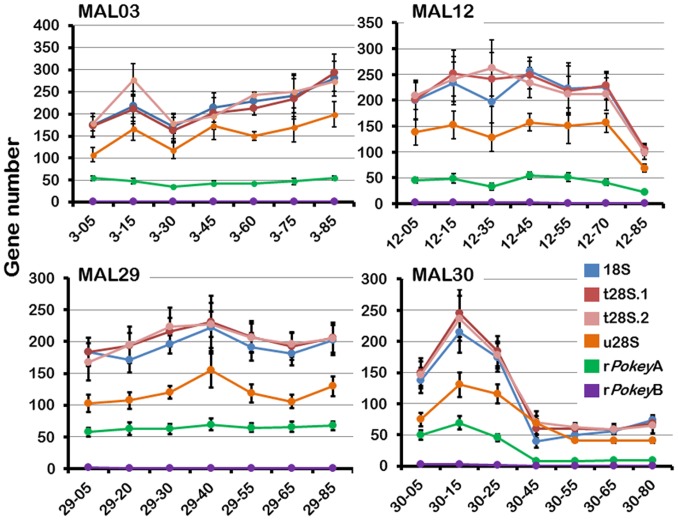
Change in rRNA gene and *Pokey* number over time in four mutation accumulation lines (MAL) of *Daphnia obtusa*. These lines were analyzed at seven time points between generation 5 and generation 85. Isolates are coded according to their line number and generation. Vertical black bars are one standard deviation. 18S = 18S rRNA genes, t28S  =  total 28S rRNA genes, u28S  =  uninserted 28S rRNA genes, r*Pokey*A  =  *Pokey*A elements in rDNA, r*Pokey*B  =  *Pokey*B elements in rDNA. t28S.1 and t28S.2 are duplicate estimates of the total number of 28S genes.

**Figure 4:**
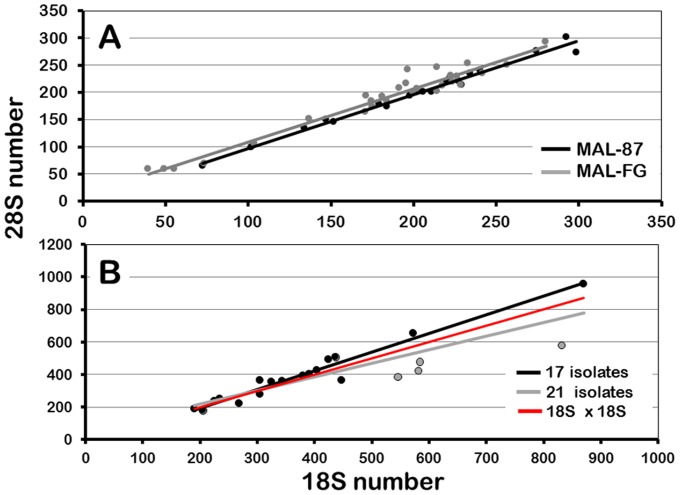
Regression of 18S and 28S rRNA gene number in *Daphnia obtusa*. (**A**) Isolates from mutation accumulation lines. The black line corresponds to the 20 lines sampled at generation ∼87 (MAL-87). The gray line corresponds to the four lines sampled at seven time points between generation 5 and 85 (MAL-FG, 28 isolates). (**B**) Isolates from natural populations in the eastern and midwestern USA. The gray line was obtained by analyzing all 21 isolates. The black line was obtained after excluding four isolates (gray points) with very low t28S number that were run on the same qPCR plate (see text). The red line was generated by plotting the number of 18S genes on both axes.

The haploid number of 18S and total (t)28S genes fluctuated between generations in all four fine-grained MAL (MAL-FG; Fig. 3, S1 Table in S1 File, S2 Figure in S3 File). For example, 18S number rose from 137 to 215 between generations 5 and 15 in MAL30, but then decreased dramatically to 40 by generation 45 and remained low thereafter. Gene number in MAL12 changed in both directions over time but by generation 85, this line had about half as many rRNA genes as it had at generation 5 (Fig. 3, S1 Table in S1 File). Conversely, MAL3 ended up with about 60% more genes than it had at generation 5. MAL29 showed the lowest variation through time, with between 181 to 223 18S genes over the 85 generations. As expected, the number of 18S and 28S genes changed in concert within each line (Fig. 3; Fig. 4A) and only two of the differences within isolates are significant after sequential Bonferroni correction (3–60 and 30–80, S6 Table in S2 File).

Haploid rRNA gene number also varied across the 20 MAL isolates sampled at ∼ generation 87 (MAL-87). 18S ranges from 73 to 299 while 28S ranges from 65 to 302 (Table 1, S1 Table in S1 File, S3 Figure in S3 File), and these values are strongly correlated within isolates with a regression line slope very close to 1 (Table 2, Fig. 4A, S9 Table in S2 File). Only three differences between 18S and 28S in the MAL-87 are significant after sequential Bonferroni correction (21–90, 30–80, 47–83, S3 Figure in S3 File, S6 Table in S2 File).

**Table 1:** Haploid number of *Pokey* and rRNA genes in isolates of *Daphnia obtusa*.

	Number of							Frequency of		
Group	Isolates	18S	t28S^3^	u28S^4^	rPokA^5^	rPokB^6^	rInserts^7^	u28S^8^	*Pokey* 9	rInserts^10^
MAL-87^1^	20	201.4^2^	197.3	130.4	56.1	1.6	9.3	0.653	0.290	0.064
		72.5 to 298.5	64.5 to 301.5	28.5 to 204.5	9 to 134	0.5 to 4	−24 to 71	0.442 to 0.873	0.103 to 0.569	−0.113 to 0.411
										
Natural	21	422.5	405.2	296.8	74.8	8.8	24.8	0.736	0.212	0.052
Populations		190 to 870.5	179.5 to 959	137.5 to 659	15 to 154	0.5 to 50.5	−155 to 149	0.508 to 0.979	0.063 to 0.343	−0.267 to 0.303

1 one isolate from each of 20 mutation accumulation lines sampled at ∼ generation 87.

2 the upper row is the mean, the lower row is the range.

3 total 28S rRNA genes.

4 28S rRNA genes without an insert in the *Pokey* TTAA insertion site.

5 *Pokey*A in 28S rRNA genes.

6 *Pokey*B in 28S rRNA genes.

7 calculated as [t28S - (u28S + r*Pokey*A + r*Pokey*B)].

8 calculated as (u28S/t28S).

9 calculated as [r*Pokey*A + r*Pokey*B)/t28S].

10 calculated as (rInserts/t28S).

**Table 2:** Correlation between the number of rRNA genes and *Pokey* elements in isolates of *Daphnia obtusa*.

Isolates	X-axis	Y-axis	n^8^	Slope	R^2^	p-value	Figure
Natural populations	18S	t28S^4^	21	0.83	0.779	1.2E-07	4
	18S	t28S	17	1.15	0.958	9.4E-12	4
	t28S	u28S^5^	21	0.70	0.803	4.0E-08	6
	t28S	rPokA^6^	21	0.16	0.542	1.4E-04	6
	t28S	rPokB^7^	21	-0.005	0.005 9	0.757	6
MAL-87^1^	18S	t28S	20	0.99	0.983	1.7E-17	4
	t28S	u28S	20	0.67	0.757	6.2E-07	6
	t28S	rPokA	20	0.38	0.516	3.6E-04	6
	t28S	rPokB	20	0.004	0.093	0.192	6
MAL-FG^2^	18S	t28S	28	0.98	0.953	8.6E-19	—
all MAL^3^	t28S.1	t28S.2	44	0.96	0.870	3.0E-20	S5
MAL 3	t28S	18S	7	0.87	0.961	1.0E-04	S10
	t28S	u28S	7	0.66	0.780	0.008	S10
	t28S	rPokA	7	0.06	0.322	0.184	S10
MAL12	t28S	18S	7	0.9	0.874	0.002	S10
	t28S	u28S	7	0.57	0.838	0.004	S10
	t28S	rPokA	7	0.14	0.408	0.123	S10
MAL29	t28S	18S	7	0.89	0.759	0.011	S10
	t28S	u28S	7	1.02	0.841	0.004	S10
	t28S	rPokA	7	0.15	0.557	0.054	S10
MAL30	t28S	18S	7	0.91	0.984	1.2E-05	S10
	t28S	u28S	7	0.46	0.879	0.002	S10
	t28S	rPokA	7	0.36	0.959	1.2E-04	S10

1 Mutation accumulation lines (MAL) sampled at ∼generation 87.

2 all isolates from the four MAL sampled at 7 time points across 85 generations.

3 all 44 isolates sampled from the MAL.

4 total 28S rRNA genes.

5 28S rRNA genes without an insert in the *Pokey* TTAA insertion site.

6 *Pokey*A in 28S rRNA genes.

7 *Pokey*B in 28S rRNA genes.

8 number of isolates in the analysis.

9 underlined values are not significant at p = 0.05 after Bonferroni correction of p-values.

If changes in rRNA gene number occur randomly within MAL, the average across lines at any given generation is expected to approximate the number of genes that were present in the progenitor female, which we estimate to have been 174 for 18S and 177 for 28S (S1 Table in S1 File) based on the mean rRNA gene number in the four MAL-FG at generation 5. Thus, there appears to have been a slight overall increase in rRNA gene number in the MAL-87 in which the mean gene numbers are 201 for 18S and 197 for 28S (Table 1).

**Figure 5:**
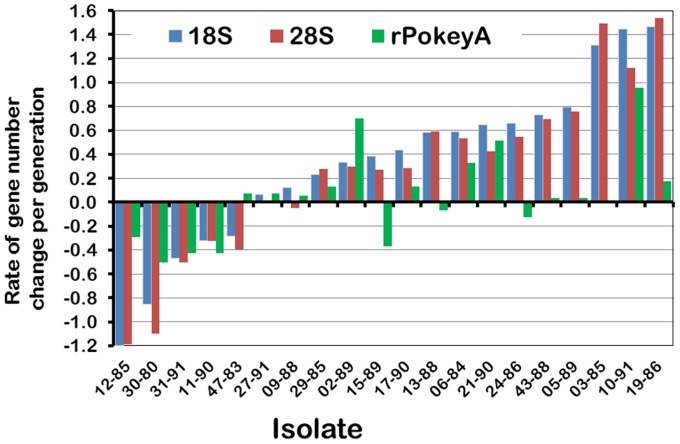
Rate of gene number change per generation in 20 mutation accumulation lines (MAL) of *Daphnia obtusa*. Gene number was only estimated once in the MAL sampled at ∼87 generations. Gene number in the progenitor female was estimated as the mean number of genes in the four MAL isolates sampled at generation 5.

Overall, rates of change per generation range from −1.2 to 1.5 copies for 18S, −1.9 to 1.5 copies for 28S and −0.5 to 1 copy for r*Pokey*A (Fig. 5, S5 Table in S2 File). The maximum rate of increase is 9.5 28S per generation over 10 generations in MAL30, and the maximum rate of decrease is −6.75 18S per generation over 20 generations in MAL30. The mean rate of rRNA gene number change across the MAL-87 is positive but less than 1 copy per generation (0.33 for 18S and 0.26 for 28S, S5 Table in S2 File).

The range of variation in haploid rRNA gene number is much wider across the 21 isolates from natural populations (NP isolates) compared to the MAL, although no NP isolate has less than 150 copies of either gene (Table 1, S1 Table in S1 File, S6 Figure in S3 File). 18S ranges from 190 to 871 with a mean of 423, and 28S ranges from 180 to 959 with a mean of 405 (Table 1). The number of 18S and t28S genes is significantly different in six isolates (GA1.2, IL3, IN1, MO2, PA, SC1.1) after Bonferroni correction (S7 Table in S2 File) and 18S is greater than t28S in all of these cases. The mean difference between 18S and t28S is 17.3 (range = −88.5 to 251), and the mean absolute difference is 63.4 (15% of mean 18S number) with a range of 1 to 251 genes. Based on the analysis of duplicate t28S estimates described below, most of these differences are likely due to experimental error during qPCR. For example, t28S is significantly lower than 18S (20% or more) in four NP isolates (IL3, IN1, MO2 and PA) that were all run on the same qPCR plate (S6 Figure in S3 File). Despite these differences, 18S and 28S are highly correlated in the NP isolates with a regression line slope of 0.83 (Table 2, Fig. 4B, S9 Table in S2 File). Both the correlation and the regression line slope (1.15) increase when the four anomalous isolates are excluded from the analysis.

### Variation in *Pokey* number

The TED analysis of *Pokey*A detected only one 237 base-pair (bp) amplicon in all 20 samples analyzed from the four MAL-FG (S10 Table). This is the amplicon we expect to be generated by *Pokey*A insertions in the 28S gene (r*Pokey*A) due to the *Bfa*I restriction site (CTAG) starting 79 nt downstream of the TTAA insertion site. Thus, we were not able to detect *Pokey*A outside of rDNA in these four lines. In addition, the 19 NP isolates all contain the 237 bp amplicon but only five contain additional amplicons (overall mean = 0.47, range = 0 to 4 excluding the 237 bp amplicon, S10 Table in S2 File).

qPCR showed that r*Pokey*A fluctuates within each of the four MAL-FG (Fig. 3, S1 Table in S1 File, S7 Figure in S3 File), as is the case for rRNA genes. For example, r*Pokey*A drops substantially after generation 25 in MAL30, along with both rRNA genes. The average haploid number of r*Pokey*A in the 20 MAL-87 isolates is 56 with values ranging from 9 to 134 (Table 1, S1 Table in S1 File, S8 Figure in S3 File), and the average rate of change is positive but very small at 0.05 copies per generation (Fig. 5, S5 Table in S2 File). r*Pokey*A in the NP isolates ranges from 15 to 154 with a mean of 75 (Table 1, S1 Table in S1 File, S9 Figure in S3 File).

On average, isolates in the four MAL-FG contain only one or two copies of r*Pokey*B, which is similar to the mean of 1.6 for the 20 MAL-87 (Table 1). Even so, r*Pokey*B persisted in the rDNA of all MAL for at least 85 generations despite its low copy number in the progenitor female. r*Pokey*B is also low in the NP isolates with a mean of 9 and a maximum of 51 (Table 1, S1 Table in S1 File, S9 Figure in S3 File). Because r*Pokey*B is very low, we subsequently designed qPCR primers to amplify all *Pokey*B (S4 Table in S2 File). Preliminary estimates of total *Pokey*B in six NP isolates are similar to, or even less than estimates of r*Pokey*B suggesting that these isolates contain few or no *Pokey*B outside of rDNA (data not shown).

**Figure 6:**
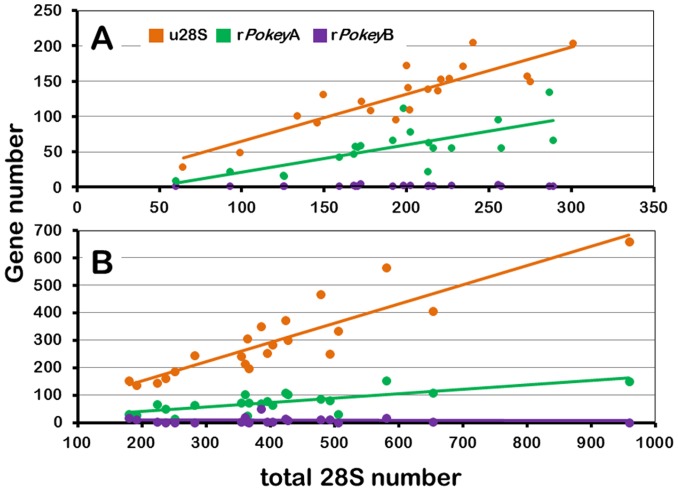
Regression of *Pokey* and 28S rRNA gene number in isolates of *Daphnia obtusa*. (**A**) Isolates from 20 mutation accumulation lines sampled at generation ∼87 (MAL-87). (**B**) Twenty-one isolates from natural populations (NP) in the eastern and Midwestern USA. u28S  =  uninserted 28S genes, r*Pokey*A  =  *Pokey*A elements in rDNA, r*Pokey*B  =  *Pokey*B elements in rDNA.

We plotted the relationship between u28S, r*Pokey*A and r*Pokey*B relative to t28S for the MAL-87 and NP isolates (Table 2, Fig. 6). There is a highly significant correlation between t28S and u28S in both groups but the regression line slopes are substantially less than 1 (Table 2, S9 Table in S2 File). There is also a significant positive correlation between t28S and r*Pokey*A in both groups, although the R^2^ values and regression line slopes are lower than those for u28S (Table 2, Fig. 6, S9 Table in S2 File). Conversely, there is no correlation between t28S and r*Pokey*B, which is not surprising given the latter's very low copy number. The average frequency of 28S genes that lack an insert in the TTAA site is 65% in the MAL-87 and 74% in the NP isolates (Table 1).

We also plotted the relationship between 18S, u28S and r*Pokey*A relative to t28S within each of the four MAL-FG (Table 2, S10 Figure in S3 File). As expected, t28S is significantly correlated with 18S across the seven time points in three lines after Bonferroni correction; the correlation is not significant in MAL29, which shows the lowest level of variation in rRNA gene number across generations (Fig. 3). t28S is also significantly correlated with u28S across the seven time points in three lines after Bonferroni correction; the correlation is not significant in MAL3 although the pattern of change for the two genes is similar in all four lines (Fig. 3, S9 Table in S2 File). While there is a positive correlation between t28S and r*Pokey*A in all four lines, it is only significant in MAL30 (Table 2, S9 Table in S2 File), in which all gene numbers decreased substantially after generation 25.

### Variation in duplicate qPCR estimates of gene number

t28S was measured twice (t28S.1 and t28S.2) in all 44 MAL isolates (28 MAL-FG and 16 MAL-87), which allowed us to evaluate the consistency of our qPCR results (S1 Table, S4 Figure in S3 File). The two estimates are not significantly different from one another in all but six isolates after sequential Bonferroni correction (3–15, 3–60, 47–83, 13–88, 21–90, 24–86, S8 Table in S2 File, S4 Figure in S3 File) and thus are highly correlated with one another (Table 2, S9 Table in S2 File, S5 Figure in S3 File). The average difference between t28S.1 and t28S.2 across all MAL isolates is 1 as the values are both positive (t28.1>t28S.2) and negative (t28S.2>t28S.1). The mean absolute difference between the two estimates is 14.6 (7.5% of t28S.1 number) with a range of 0 to 66 (0 to 31%).

Assuming that *Pokey* elements are the only sequences that insert into the TTAA site between our u28S qPCR primers, we expect that t28S would approximately equal the sum of (u28S + r*Pokey*A + r*Pokey*B). Eagle and Crease [28] referred to the difference between t28S number and this sum, which can be either positive or negative, as rInserts. We estimated the average number of rInserts to be 9 in the MAL-87, which is 6.4% of t28S, and 25 in the NP isolates, which is 5.2% of t28S (Table 1). The range of values is similar to the range of differences between the duplicate estimates of t28S in MAL isolates, and there is no correlation between the number of rInserts and any of t28S, u28S or r*Pokey*A in the NP isolates (R^2^ = 0.004 to 0.06, p>0.25).

## Discussion

### Variation in duplicate qPCR estimates of gene number

Variation between duplicate estimates of t28S in the 44 MAL isolates is as high as 30%. However, the average is 7.5%, and there is no apparent bias in terms of which estimate (the first or the second) is higher. Indeed, the average difference overall is only one gene copy. We also observed substantial discrepancies in some isolates between the total number of 28S genes and rInserts [u28S + r*Pokey*A + r*Pokey*B], which is expected to be zero if no other TEs insert between the u28S primer sites. Eagle and Crease [28] found that the number of rInserts was overwhelmingly positive and significantly positively correlated with t28S in *D. pulex* and *D. pulicaria* suggesting that insertions other than *Pokey* could be present. Although the absolute number of rInserts is quite large in some of the *D. obtusa* isolates from natural populations (Table 1), we obtained both positive and negative estimates (mean = 24.8, which is 6% of mean t28S) and there is no correlation between rInserts and the number of genes that contribute to their value (i.e. t28S, u28S, r*Pokey*A). Overall, this suggests that *D. obtusa* rInserts are most likely a product of experimental variation generated during qPCR, and there is little or no bias in the tendency of our qPCR primers to over- or underestimate the number of genes they amplify, which is consistent with the results we obtained for the replicate estimates of t28S.

Despite the variation we observed in gene number estimates, the basic pattern of variation across isolates is not likely to be obscured, and indeed, positive relationships that we expected to occur (e.g. correlation between 18S and t28S, and between t28S and u28S) are highly significant (Table 2). Moreover, the highly significant positive correlation between r*Pokey*A and t28S in NP isolates is clearly not an artifact. Indeed, the error we are most likely to make because of “noisy” qPCR data is failure to identify a weak, but significant correlation between the numbers of different genes. In fact, this may have occurred in two of the MAL-FG where the correlation between r*Pokey*A and t28S is nearly significant (Table 2). Even if this is the case, it does not change the fact that r*Pokey*A is not as strongly correlated with t28S as is u28S.

### Variation in rRNA gene number

We detected substantial variation in the number of rRNA genes in mutation accumulation lines propagated by apomictic reproduction, on which natural selection is relaxed. Despite substantial variation, the average rate of change in 18S is less than 1 gene per generation suggesting that the direction of change is not substantially biased either up or down under relaxed selection. As expected, t28S is very similar to 18S and few of the differences are significant after Bonferroni correction. Averbeck and Eickbush [30] measured rRNA gene number in 16 MAL of *Drosophila melanogaster* after 400 generations and found that it varied from 140 to 310, which is similar to the level of variation we observed in the *D. obtusa* lines (65 to 302), although the *Daphnia* variation was generated in only 87 generations.

We also detected substantial variation in rRNA gene number in isolates of *D. obtusa* from natural populations (Table 1), which are subject to the operation of natural selection. However, both the mean and range of rRNA gene number is much greater among the 21 NP isolates (18S mean = 423, range = 190 to 871; 28S mean = 405, range = 180 to 959) than among the MAL-87 (18S mean = 201, range = 73 to 299; 28S mean = 197, range = 65 to 302). Moreover, no values below 150 were observed for either gene in the NP isolates, suggesting that the rDNA locus of the MAL progenitor female (∼175 genes) was at the low end of the size distribution in nature.

*D. pulex* and *D. pulicaria* are members of the *D. pulex* species complex and diverged from one another within the last 2 million years, but diverged from *D. obtusa* on the order of 50 million years ago [31]. Eagle and Crease [28] observed a somewhat different pattern for the rRNA genes in 69 isolates of these two species from natural populations in eastern and midwestern North America compared to *D. obtusa*. 18S averaged about 220 in both species (range of 94 to 490) and 28S averaged about 260 (range of 88 to 725), which is 48% less than the average rRNA gene number we observed in NP isolates of *D. obtusa*.

Simulation studies by Zhang et al. [11] and Zhou et al. [12] suggest that relative rates of sister chromatid and interchromosomal exchange have a substantial effect on the size of the rDNA locus. Interchromosomal exchange tends to decrease both the overall size of the locus and copy number variation among individuals, while sister chromatid exchange does the opposite; higher recombination rates lead to larger loci and higher levels of interindividual variation. The wide range of rRNA gene number in all three *Daphnia* species suggests that sister chromatid exchange occurs at a higher rate than interchromosomal exchange, which is consistent with indirect evidence obtained in previous studies of *D. pulex* [25], [32], [33]. Moreover, McTaggart et al. [25] estimated the rate of rDNA recombination in the *D. obtusa* MAL to be 0.02 to 0.06 events per generation, which is at the high end of values reported for other organisms (10^−2^ to 10^−5^, [25] and references within). Based on the simulation results [11], [12] and the smaller size of the rDNA locus in *D. pulex* and *D. pulicaria*, we predict that the rate of rDNA recombination in these two species is substantially lower than the rate in *D. obtusa*. We are now looking for isolates of *D. pulex* or *D. pulicaria* that contain sufficient intraindividual rDNA variation to allow a test of this prediction.

### Variation in *Pokey* number

We found that r*Pokey*A varies substantially among the 20 MAL-87 isolates and is significantly correlated with t28S (Table 2, Fig. 6). Although a similar pattern was observed through time in the MAL-FG, copy number fluctuations were not substantial in some lines and the correlation between t28S and r*Pokey*A is only significant in MAL30, in which there was a substantial loss of all genes after generation 25 (Fig. 3). In contrast, r*Pokey*B is very low (1 to 2 copies per haploid genome) in the four MAL-FG and in all lines after 87 generations, although it was never eliminated. On average, 35% of the 28S genes in MAL isolates contain an r*Pokey* element, or failed to amplify with our u28S primers (Table 1), which is very similar to the patterns observed by Averbeck and Eickbush [30] for the non-LTR retrotransposons, R1 and R2 in *D. melanogaster* MAL. R1 number (∼50 to 100) was higher than R2 number (stable at ∼25 copies) across the 16 lines they studied. Moreover, R1 was strongly correlated with rDNA locus size, while R2 was not. On average, 55% of 28S genes in an individual contained a copy of either R1 or R2 (or both) after 400 generations.

The study by Averbeck and Eickbush [30] and subsequent empirical studies on patterns of R2 and rDNA transcription in *Drosophila* led Eickbush et al. [34] to propose the domain model of R2 transcription regulation, which was expanded to consider the population dynamics of R-elements by Zhou et al. [12]. In this model, 28S genes containing R2 insertions serve as foci for heterochromatization, which spreads to neighboring rDNA units. As a result, transcriptionally active rDNA generally consists of a contiguous block of rDNA units with the lowest level of R-element insertions, which they called the transcription domain. Moreover, the size of these transcription domains will vary as a consequence of recombination, as does the size of the entire rDNA locus [11]. As long as the transcription domain is free of R-elements, the elements will not be transcribed and will remain inactive. Conversely, if contiguous blocks of insert-free rDNA units are shorter than a transcription domain, and/or R-elements are highly dispersed, the transcription domain may contain one or more R-elements, which can then be transcribed and subsequently transpose.

It has also been observed that recombination events in *Drosophila* rDNA tend to occur in transcriptionally active regions ([30] and references within). Thus, R-elements that are clustered in inactive rDNA regions will generally not be involved in the recombination events that change rDNA copy number, while those that are dispersed and/or occur in transcription domains will. Overall, this suggests that R2, whose numbers were low and stable in the *Drosophila* MAL studied by Averbeck and Eickbush [30], was more clustered in the rDNA array than R1.

Despite the fact that *Pokey* is a DNA transposon with a different transposition mechanism than the non-LTR R-elements, the transcription domain model may also explain the pattern we observed with r*Pokey*A and r*Pokey*B in the *D. obtusa* MAL. Like R2, r*Pokey*B is present in very low but stable numbers and was neither eliminated nor propagated over 87 generations despite substantial fluctuations in t28S number. Conversely, r*Pokey*A occurs in high number (mean = 56, range = 9 to 134) and fluctuates with t28S, although not to the same extent as u28S. These patterns suggest that r*Pokey*B copies are clustered and typically reside in regions that have low rDNA recombination rates and are rarely transcriptionally active, which leads to low rates of transcription (and transposition) and little proliferation by recombination. Conversely, r*Pokey*A copies are likely to be more dispersed and occur in regions that have higher recombination rates. Thus, they may frequently be involved in the recombination events that change their number and the number of rRNA genes. In addition, they may be more transcriptionally active, providing opportunities for transposition that can also lead to increased copy number. Although the number of 28S genes with *Pokey* inserts is higher in the NP isolates, the average proportion of inserted genes is lower at 26% compared to 35% in the MAL-87 isolates (Table 1). This is consistent with the expectation that expansion of TEs beyond a certain point would be opposed by natural selection. If the domain model of TE transcription regulation in rDNA is correct, one hypothesis to explain why *Pokey* successfully colonized rDNA, but other DNA TEs have not is that *Pokey* contains sequences that attract rDNA heterochromatization complexes. Future work could be aimed at identifying such sequences.

The results of this study differ considerably from those obtained by Eagle and Crease [28] for the 69 isolates of *D. pulex* and *D. pulicaria* in which r*Pokey*A averaged only four copies per haploid genome (range = 0 to 45). The fact that r*Pokey*A is much more frequent (mean = 75, range = 15 to 154) and strongly correlated with t28S in *D. obtusa* but not correlated in *D. pulex* and *D. pulicaria* suggests that r*Pokey*A tends to be clustered in the rDNA of the latter two species, which is consistent with previous results obtained for *D. pulex* by Glass et al. [35]. As discussed above, such clustering of *Pokey* in rDNA would inhibit its spread by both transposition (transcriptionally inactive) and recombination. Overall, we suggest that an increased rate of sister chromatid exchange and a more dispersed distribution of r*Pokey*A explains both the larger rDNA locus and much higher number of r*Pokey*A in *D. obtusa* compared to *D. pulex* or *D. pulicaria*. Even if 40% of the 400 28S genes per haploid genome in an average *D. obtusa* individual contain a *Pokey* insertion, the number of u28S genes would still exceed 200. Two hundred is the about the same size as the average rDNA locus in *D. pulex* and *D. pulicaria*, which generally have very few r*Pokey* insertions.

Although r*Pokey*A number is generally higher in the NP than the MAL isolates, g*Pokey*A number may be very low in both groups. We only detected TED amplicons outside rDNA in 5 of the 19 NP isolates and in none of the MAL isolates. It is possible that g*Pokey*A was not efficiently amplified in the TED analysis due to interference from the high number of rDNA copies, in which case we could have under-estimated the number of g*Pokey*A. Even if this is the case, it is clear that there are substantially more *Pokey*A inside than outside of rDNA. The preliminary results based on qPCR also indicate that *Pokey*B is rare or even absent outside of rDNA in *D. obtusa*. This is substantially different from the distribution of *Pokey* in *D. pulex* and *D. pulicaria* in which average g*Pokey*A is only 10 copies per haploid genome (range = 4 to 24), but this represents more than 70% of the total *Pokey*A insertions in these two species [28].

## Conclusions

Our results suggest that there are complex interactions between the two related, but divergent *Pokey* elements and rDNA in *Daphnia* genomes. For example, the high recombination rate and consequently larger rDNA locus size may have facilitated the expansion of r*Pokey*A to higher number in *D. obtusa* than in *D. pulicaria* and *D. pulex*, whose rDNA loci are about half as large and generally contain very few r*Pokey* insertions. In addition, r*Pokey*A in *D. pulex* and *D. pulicaria* tend to be clustered while the strong correlation with 28S suggests that they are dispersed in *D. obtusa*. Moreover, preliminary analyses suggest that *Pokey* is much more common inside than outside rDNA in *D. obtusa* while the reverse is true in *D. pulex* and *D. pulicaria*.

R-elements have been stable occupants of arthropod rDNA for hundreds of millions of years, but they are retroelements with substantial rates of replicative transposition [30]. Conversely, *Pokey* is a DNA transposon, the life cycles of which are generally thought to end with inactivation by the host over time [3]. Despite this major difference in TE life history, patterns of r*Pokey*A and r*Pokey*B distribution are similar to those of R1 and R2 in *Drosophila*, and consistent with the domain model of R-element transcription regulation proposed by [34]. Thus, *Pokey*'s successful colonization of rDNA, which is a unique niche for a DNA transposon, may have facilitated its long-term persistence and activity in the subgenus *Daphnia*.

## Supporting Information

S1 File Contains the following files: **S1 Table.** *Pokey* and rRNA gene number in isolates of *Daphnia obtusa* analyzed in this study.**S2 Table.** C_T_ values from qPCR analysis of *Pokey* and rRNA gene number in isolates of *Daphnia obtusa* analyzed in this study.
(XLSX)

S2 File Contains the following files: **S3 Table.** In Primers and linkers used for Transposable Element Display of *Pokey*A in *Daphnia obtusa*. **S4 Table.** Primers for qPCR of rRNA gene and *Pokey* number in *Daphnia obtusa*. **S5 Table.** Rate of gene number change in mutation accumulation lines of *Daphnia obtusa* over ∼87 generations. **S6 Table.** In S2 File. Bonferroni correction of p-values from pairwise t-tests of differences between 18S and total 28S rRNA gene number in 44 isolates of *Daphnia obtusa* from mutation accumulation lines. **S7 Table.** Bonferroni correction of p-values from pairwise t-tests of differences between 18S and total 28S rRNA gene number in 22 isolates of *Daphnia obtusa* from natural populations. **S8 Table.** Bonferroni correction of p-values from pairwise t-tests of differences between duplicate estimates of total 28S rRNA genes (t28S.1 and t28S.2) in 44 isolates of *Daphnia obtusa* from mutation accumulation lines. **S9 Table.** Bonferroni correction of p-values from regression analyses in Table 2. **S10 Table.** number in isolates of *Daphnia obtusa* from natural populations based on Transposable Element Display.
(PDF)

S3 File Contains the following figures: **S1 Figure.** Distribution of *Tif*:*Gtp* ratios in qPCR analysis of rRNA gene and *Pokey* copy number in *Daphnia obtusa*. **S2** **Figure.** Number of 18S and 28S rRNA genes in isolates from 4 fine-grained mutation accumulation lines of *Daphnia obtusa* sampled at 7 time points between generation 5 and 85. **S3 Figure.** Number of 18S and 28S rRNA genes in isolates from 20 mutation accumulation lines of *Daphnia obtusa* sampled at generation ∼87. **S4 Figure.** Duplicate estimates of total 28S rRNA number gene (t28S.1, t28S.2) in all 44 isolates from *Daphnia obtusa* mutation accumulation lines. **S5 Figure.** Regression of duplicate estimates of t28S number (t28S.1, t28S.2) in all 44 isolates from *Daphnia obtusa* mutation accumulation lines. **S6 Figure.** Number of 18S and 28S rRNA genes in *Daphnia obtusa* isolates from natural populations. **S7 Figure.** Number of *Pokey* and 28S rRNA genes without inserts (u28S) in *Daphnia obtusa* isolates from 4 fine-grained mutation accumulation lines sampled at 7 time points between generation 5 and 85. **S8 Figure.** Number of *Pokey* and 28S rRNA genes without inserts (u28S) in *Daphnia obtusa* isolates from 20 mutation accumulation lines sampled at generation ∼87. **S9 Figure.** Number of *Pokey* and 28S rRNA genes without inserts (u28S) in *Daphnia obtusa* isolates from natural populations. **S10 Figure.** Regression of rRNA gene and r*Pokey*A number in 4 fine-grained *Daphnia obtusa* MAL sampled at 7 time points between generation 5 and 85.
(PDF)
