# Supplementary material for: Copy Number of the Transposon, Pokey, in rDNA Is Positively Correlated with rDNA Copy Number in Daphnia obtusa
Source: PLoS One. 2014 Dec 9;9(12):e114773. doi: 10.1371/journal.pone.0114773 (PMC4260951; doi:10.1371/journal.pone.0114773)
Supplement: S2 File — Contains the following files: S3 Table. In Primers and linkers used for Transposable Element Display of PokeyA in Daphnia obtusa. S4 Table. Primers for qPCR of rRNA gene and Pokey number in Daphnia obtusa. S5 Table. Rate of gene number change in mutation accumulation lines of Daphnia obtusa over ∼87 generations. S6 Table. In S2 File. Bonferroni correction of p-values from pairwise t-tests of differences between 18S and total 28S rRNA gene number in 44 isolates of Daphnia obtusa from mutation accumulation lines. S7 Table. Bonferroni correction of p-values from pairwise t-tests of differences between 18S and total 28S rRNA gene number in 22 isolates of Daphnia obtusa from natural populations. S8 Table. Bonferroni correction of p-values from pairwise t-tests of differences between duplicate estimates of total 28S rRNA genes (t28S.1 and t28S.2) in 44 isolates of Daphnia obtusa from mutation accumulation lines. S9 Table. Bonferroni correction of p-values from regression analyses in Table 2. S10 Table. number in isolates of Daphnia obtusa from natural populations based on Transposable Element Display. (PDF) [file pone.0114773.s002.pdf]

Table S3.

Primers and linkers used for Transposable Element Display of *Pokey* A in *Daphnia obtusa*.

**Primer/Linker**	**Sequence 5'-3'**	**Purpose**
Bfa I Forward Linker	tac tca gga ctc at	ligation
Bfa I Reverse Linker	gac gat gag tcc tga g	ligation
Bfa I Reverse Primer	gac gat gag tcc tga gta g	primary and secondary PCR
PokA6456F	gac aac ggt ggc cga aac gcg g	primary PCR
6Fam-PokA6464 F	tgg ccg aaa cgc ggt tag gcc g	secondary PCR

Table S4. Primers for qPCR of rRNA gene and *Pokey* number in *Daphnia obtusa.*

Fragment	Primer	Sequence 5' to 3'	Purpose	Amplicon size (bp)	Threshold[1]	PAE
18S	18S1864 F	ccg cgt gac agt gag caa ta	total 18S	50	0.2	0.95
	18S1913 R	ccc agg aca tct aag ggc atc				
t28S	28S2508 F	gcc tgc tcg tac cga tat cc	total 28S	50	0.2	0.94
	28S2558 R	cta gag gct gtt cac ctt gga ga				
u28S	28S3023 F	taa acg gcg gga gta act atg ac	28S without inserts	50	0.2	0.94
	28S3073 R	tga cga ggc att tgg cta cc				
rPokeyA	PokA6456F	gac aac ggt ggc cga aac gcg g	*Pokey* A in rDNA	171	0.326	0.94
	28S3073 R	tga cga ggc att tgg cta cc				
rPokeyB	PokB4283 F	aat ttc agt caa gca cgg cc	*Pokey* B in rDNA	70	0.244	0.91
	28S3073 R	tga cga ggc att tgg cta cc				
tPokeyB	PokB4283 F	aat ttc agt caa gca cgg cc	*Pokey* B in rDNA	47	0.2	not
	PokB4330R	aac cct ttt tcg acg cca aag	and other genomic locations			determined
GTP	GTP385 F	tat tca gca tgg aga gac ggc	single copy control	50	0.2	0.93
	GTP435 R	gat gtc gac tga cgc tgg aa				
TIF	TIF392 F	gac atc atc ctg gtt ggc ct	single copy control	50	0.2	0.94
	TIF442 R	aac gtc agc ctt ggc atc tt				

1. The threshold for estimating the C_T value of 50-bp qPCR amplicons was set to 0.2. The threshold of longer amplicons was adjusted according to the formula 0.2 × 2^[1-(50/amplicon length in bp)] as in Eagle and Crease (2012).

Table S5. Rate of gene number change in Mutation Accumulation lines of Daphnia obtusa over ~ 87 generations

Gene number was only estimated once in the MAL-87s, so we used the average number of genes in the four MAL-FGs at generation 5 to estimate gene number in the progenitor female. The mean gene numbers are 174 for 18S, 177 for 28S and 52 for rPokeyA.

	copy number change per generation		
**MAL**	**18S**	**28S**	**rPokeyA**
12-85	-1.20	-1.19	-0.29
30-80	-0.85	-1.10	-0.50
31-91	-0.47	-0.50	-0.42
11-90	-0.32	-0.32	-0.42
47-83	-0.28	-0.40	0.07
27-91	0.06	0.02	0.07
09-88	0.12	-0.05	0.05
29-85	0.23	0.28	0.13
02-89	0.33	0.30	0.70
15-89	0.38	0.27	-0.37
17-90	0.44	0.28	0.13
13-88	0.58	0.59	-0.07
06-84	0.59	0.53	0.33
21-90	0.65	0.42	0.51
24-86	0.66	0.54	-0.12
43-88	0.73	0.69	0.04
05-89	0.79	0.76	0.04
03-85	1.31	1.49	0.01
10-91	1.45	1.12	0.95
19-86	1.46	1.54	0.17
**mean**	**0.33**	**0.26**	**0.05**
**range**	**-1.20 to 1.46**	**-1.90 to 1.54**	**-0.50 to 0.95**

	**18S**	**28S**	**rPokeyA**
**MAL03**	+1.31 (-3.1 to 4.3)	+1.49 (-3.2 to 5.9)	+0.01 (-0.9 to 0.8)
**MAL12**	-1.2 (-8.2 to 6.0)	-1.19 (-8.2 to 5.2)	-0.29 (-1.3 to 2.1)
**MAL29**	+0.23 (-2.1 to 2.7)	+0.28 (-1.6 to 2.4)	+0.13 (-0.3 to 0.4
**MAL30**	-0.85 (-6.8 to 7.8)	-1.1 (-6.2 to 9.5)	-0.5 (-2.4 to 2.0)

Table S6. Bonferroni correction of p-values from pairwise t-tests of differences between 18S and total 28S rRNA gene number in 44 isolates of *Daphnia obtusa* from mutation accumulation lines.

isolate	p-value	corrected	result
02-89	0.9868	0.05000	not significant
05-89	0.9705	0.02500	not significant
29-05	0.9488	0.01667	not significant
31-91	0.9440	0.01250	not significant
12-05	0.9063	0.01000	not significant
06-84	0.8988	0.00833	not significant
03-05	0.8424	0.00714	not significant
13-88	0.8026	0.00625	not significant
11-90	0.7796	0.00556	not significant
27-91	0.7739	0.00500	not significant
12-55	0.7412	0.00455	not significant
12-70	0.7131	0.00417	not significant
09-88	0.6577	0.00385	not significant
30-65	0.6304	0.00357	not significant
12-85	0.6279	0.00333	not significant
29-65	0.6149	0.00313	not significant
15-89	0.5898	0.00294	not significant
03-15	0.5765	0.00278	not significant
29-85	0.5239	0.00263	not significant
29-40	0.4724	0.00250	not significant
17-90	0.4393	0.00238	not significant
03-30	0.3934	0.00227	not significant
19-86	0.3380	0.00217	not significant
12-45	0.3316	0.00208	not significant
29-55	0.2947	0.00200	not significant
03-85	0.2900	0.00192	not significant
30-05	0.2770	0.00185	not significant
24-86	0.2031	0.00179	not significant
03-75	0.1886	0.00172	not significant
30-25	0.1547	0.00167	not significant
17-90	0.1480	0.00161	not significant
12-15	0.1418	0.00156	not significant
12-35	0.0628	0.00152	not significant
30-15	0.0462	0.00147	not significant
10-91	0.0331	0.00143	not significant
03-45	0.0272	0.00139	not significant
29-20	0.0229	0.00135	not significant
29-30	0.0156	0.00132	not significant
30-45	0.0022	0.00128	not significant
30-80	0.000894	0.00125	**significant**
03-60	0.000087	0.00122	**significant**
21-90	0.000041	0.00119	**significant**
47-83	0.000031	0.00116	**significant**
30-55	0.000002	0.00114	**significant**

Table S7. Bonferroni correction of p-values from pairwise t-tests of differences between 18S and total 28S rRNA gene number in 22 isolates of *Daphnia obtusa* from natural populations.

isolate	pvalue	corrected	result
SC2	0.896	0.05000	not significant
SC1.2	0.809	0.02500	not significant
GA1.1	0.755	0.01667	not significant
IL1.2	0.711	0.01250	not significant
IL1.1	0.590	0.01000	not significant
IL2	0.375	0.00833	not significant
MO1.1	0.360	0.00714	not significant
SC3	0.296	0.00625	not significant
IL1.4	0.195	0.00556	not significant
OK1	0.095	0.00500	not significant
MO1.2	0.018	0.00455	not significant
IL1.3	0.013	0.00417	not significant
OK2	0.0088	0.00385	not significant
IN2	0.0071	0.00357	not significant
TX	0.0067	0.00333	not significant
GA1.2	0.00157	0.00313	**significant**
IL3	0.00096	0.00294	**significant**
SC1.1	0.00017	0.00278	**significant**
MO2	8.8E-05	0.00263	**significant**
PA	2.1E-06	0.00250	**significant**
IN1	6.6E-08	0.00238	**significant**

Table S8. Bonferroni correction of p-values from pairwise t-tests of differences between duplicate estimates of total 28S rRNA genes (t28S.1 and t28S.2) in 44 isolates of *Daphnia obtusa* from mutation accumulation lines.

isolate	p-value	corrected	result
30-65	0.961	0.0500	not significant
29-85	0.867	0.0250	not significant
29-55	0.839	0.0167	not significant
29-20	0.807	0.0125	not significant
09-88	0.777	0.0100	not significant
29-40	0.735	0.0083	not significant
02-89	0.651	0.0071	not significant
29-65	0.590	0.0063	not significant
12-55	0.574	0.0056	not significant
05-30	0.474	0.0050	not significant
3-05	0.473	0.0045	not significant
3-45	0.471	0.0042	not significant
12-05	0.449	0.0038	not significant
30-15	0.446	0.0036	not significant
12-15	0.419	0.0033	not significant
29-30	0.413	0.0031	not significant
27-91	0.357	0.0029	not significant
10-91	0.353	0.0028	not significant
30-25	0.338	0.0026	not significant
19-86	0.297	0.0025	not significant
17-90	0.287	0.0024	not significant
05-89	0.283	0.0023	not significant
12-35	0.237	0.0022	not significant
15-89	0.221	0.0021	not significant
3-75	0.203	0.0020	not significant
06-84	0.163	0.0019	not significant
43-88	0.162	0.0019	not significant
30-80	0.132	0.0018	not significant
31-91	0.127	0.0017	not significant
12-85	0.068	0.0017	not significant
12-70	0.061	0.0016	not significant
3-85	0.061	0.0016	not significant
12-45	0.060	0.0015	not significant
3-30	0.054	0.0015	not significant
30-55	0.039	0.0014	not significant
30-45	0.026	0.0014	not significant
05-29	0.024	0.0014	not significant
11-90	0.00296	0.00132	not significant
47-83	0.00076	0.00128	**significant**
13-88	0.00012	0.00125	**significant**
21-90	5.4E-05	0.00122	**significant**
24-86	9.1E-06	0.00119	**significant**
3-60	4.6E-06	0.00114	**significant**
3-15	3.9E-07	0.00116	**significant**

Table S9. Bonferroni correction of p-values from regression analyses in Table 2

NP = isolates from natural populations

MAL-87 = isolates from mutation accumulation lines sampled at ~generation 87

MAL-FG = four mutation accumulation lines sampled at 7 time points across 85 generations

t28S = total 28S rRNA genes

u28S = 28S rRNA genes lacking *Pokey* insertions

r*Pokey* A = *Pokey* A element in 28S genes

r*Pokey* B = *Pokey* B element in 28S genes

Isolates	x-axis	y-axis	p-value	corrected	result
NP	t28S	rPokeyB	0.7570	0.05000	not significant
MAL-87	t28S	rPokeyB	0.1920	0.02500	not significant
MAL 3	t28S	rPokeyA	0.1836	0.01667	not significant
MAL12	t28S	rPokeyA	0.1230	0.01250	not significant
MAL29	t28S	rPokeyA	0.0540	0.01000	not significant
MAL29	t28S	18S	0.0110	0.00833	not significant
MAL 3	t28S	u28S	0.0084	0.00714	not significant
MAL12	t28S	u28S	0.0040	0.00625	**significant**
MAL29	t28S	u28S	0.0040	0.00556	**significant**
MAL12	t28S	18S	0.0020	0.00500	**significant**
MAL30	t28S	u28S	0.0020	0.00455	**significant**
MAL-87	t28S	rPokeyA	3.6E-04	0.00417	**significant**
NP	t28S	rPokeyA	1.4E-04	0.00385	**significant**
MAL30	t28S	rPokeyA	0.0001	0.00357	**significant**
MAL 3	t28S	18S	0.0001	0.00333	**significant**
MAL30	t28S	18S	0.0000	0.00313	**significant**
MAL-87	t28S	u28S	6.2E-07	0.00294	**significant**
NP-21 isolates	18S	t28S	1.2E-07	0.00278	**significant**
NP	t28S	u28S	4.0E-08	0.00263	**significant**
NP-17 isolates	18S	t28Sx	9.4E-12	0.00250	**significant**
MAL-87	18S	t28S	1.7E-17	0.00238	**significant**
MAL-FG	18S	t28S	8.6E-19	0.00227	**significant**
all MAL	t28S.1	t28S.2	3.0E-20	0.00217	**significant**

Table S10. ***PokeyA*** **number in isolates of** ***Daphnia obtusa*** **from natural populations based on Transposable Element Display.** The 237 nt fragment represents *Pokey* insertions in the 28S gene and is not included in the total.
MAL-FG = fine-grained mutation accumulation lines. These values are not included in the mean.

		Fragment Size (nt)															
**Element**	**Isolate**	**177**	**208**	**227**	**237**	**241**	**242**	**244**	**247**	**254**	**256**	**267**	**301**	**315**	**421**	**518**	**Total**
Pokey A	**MAL-FG**	0	0	0	x	0	0	0	0	0	0	0	0	0	0	0	**0**
Pokey A	GA-1.1	0	0	0	x	0	0	0	0	0	0	0	0	0	0	0	**0**
Pokey A	IL-1.1	0	0	0	x	0	0	0	0	0	0	0	0	0	0	0	**0**
Pokey A	IL-1.2	0	0	0	x	0	0	0	0	0	0	0	0	0	0	0	**0**
Pokey A	IL-1.3	0	0	0	x	0	0	0	0	0	0	0	0	0	0	0	**0**
Pokey A	IL-1.4	0	0	0	x	0	0	0	0	0	0	0	0	0	0	0	**0**
Pokey A	IL-3	0	0	0	x	0	0	0	0	0	0	0	0	0	0	0	**0**
Pokey A	IN-1	0	0	0	x	0	0	0	0	0	0	0	0	0	0	0	**0**
Pokey A	IN-2	0	0	0	x	0	0	0	0	0	0	0	0	0	0	0	**0**
Pokey A	MO-1.1	0	0	0	x	0	0	0	0	0	0	0	0	0	0	0	**0**
Pokey A	MO-2	0	0	0	x	0	0	0	0	0	0	0	0	0	0	0	**0**
Pokey A	OK-1	0	0	0	x	0	0	0	0	0	0	0	0	0	0	0	**0**
Pokey A	SC-1.2	0	0	0	x	0	0	0	0	0	0	0	0	0	0	0	**0**
Pokey A	SC-2	0	0	0	x	0	0	0	0	0	0	0	0	0	0	0	**0**
Pokey A	SC-3	0	0	0	x	0	0	0	0	0	0	0	0	0	0	0	**0**
Pokey A	OK-2	0	0	0	x	1	0	0	0	0	0	0	0	0	0	0	**1**
Pokey A	PA	0	0	0	x	0	0	1	0	0	0	0	0	0	0	0	**1**
Pokey A	SC-1.1	0	0	0	x	0	0	0	0	0	0	0	0	0	0	1	**1**
Pokey A	IL-2	0	1	0	x	0	0	0	1	0	0	0	0	0	0	0	**2**
Pokey A	TX	1	0	0	x	0	1	0	1	0	0	1	0	0	0	0	**4**
Pokey A	**Total**	**1**	**1**	**0**	**19**	**1**	**1**	**1**	**2**	**0**	**0**	**1**	**0**	**0**	**0**	**1**	**0.474**
																	**mean**

**MAL-FG isolates**
03-10
03-35
03-55
03-70
03-90
12-10
12-35
12-55
12-70
12-90
29-10
29-35
29-45
29-70
29-86
30-10
30-35
30-50
30-70
30-92
