# Supplementary material for: Copy Number of the Transposon, Pokey, in rDNA Is Positively Correlated with rDNA Copy Number in Daphnia obtusa
Source: PLoS One. 2014 Dec 9;9(12):e114773. doi: 10.1371/journal.pone.0114773 (PMC4260951; doi:10.1371/journal.pone.0114773)
Supplement: S3 File — Contains the following figures: S1 Figure. Distribution of Tif:Gtp ratios in qPCR analysis of rRNA gene and Pokey copy number in Daphnia obtusa. S2 Figure. Number of 18S and 28S rRNA genes in isolates from 4 fine-grained mutation accumulation lines of Daphnia obtusa sampled at 7 time points between generation 5 and 85. S3 Figure. Number of 18S and 28S rRNA genes in isolates from 20 mutation accumulation lines of Daphnia obtusa sampled at generation ∼87. S4 Figure. Duplicate estimates of total 28S rRNA number gene (t28S.1, t28S.2) in all 44 isolates from Daphnia obtusa mutation accumulation lines. S5 Figure. Regression of duplicate estimates of t28S number (t28S.1, t28S.2) in all 44 isolates from Daphnia obtusa mutation accumulation lines. S6 Figure. Number of 18S and 28S rRNA genes in Daphnia obtusa isolates from natural populations. S7 Figure. Number of Pokey and 28S rRNA genes without inserts (u28S) in Daphnia obtusa isolates from 4 fine-grained mutation accumulation lines sampled at 7 time points between generation 5 and 85. S8 Figure. Number of Pokey and 28S rRNA genes without inserts (u28S) in Daphnia obtusa isolates from 20 mutation accumulation lines sampled at generation ∼87. S9 Figure. Number of Pokey and 28S rRNA genes without inserts (u28S) in Daphnia obtusa isolates from natural populations. S10 Figure. Regression of rRNA gene and rPokeyA number in 4 fine-grained Daphnia obtusa MAL sampled at 7 time points between generation 5 and 85. (PDF) [file pone.0114773.s003.pdf]

Figure S1. Distribution of Tif:Gtp ratios in qPCR analysis of rRNA gene and *Pokey* copy number in *Daphnia obtusa*.
The histogram shows TG values corresponding to ratios (from left to right) of 2:4, 2:3 and 2:2. The mean TG ratio for values corresponding to a 2:2 ratio is 0.77.

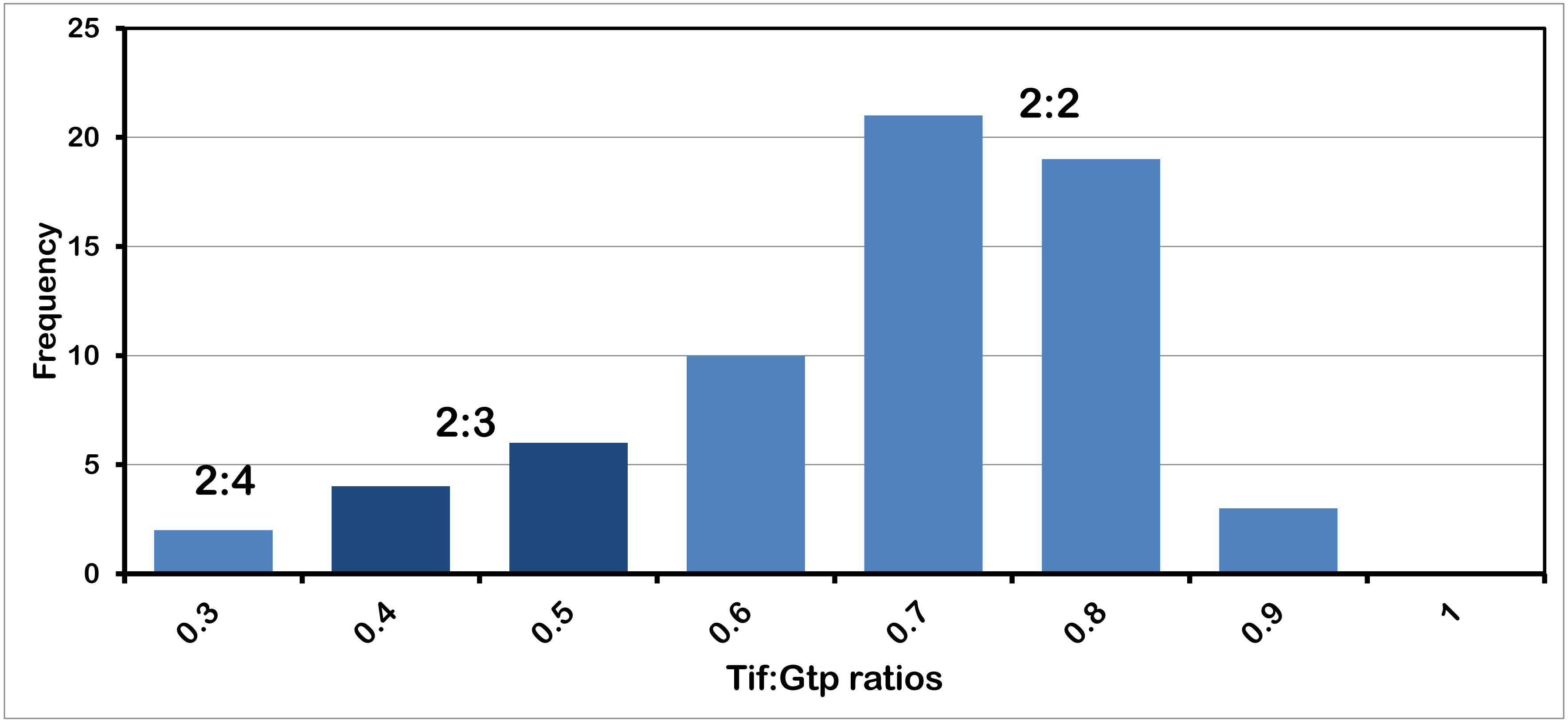

Bin	Frequency
0.3	2
0.4	4
0.5	6
0.6	10
0.7	21
0.8	19
0.9	3
1	0
	**65**

Figure S2. Number of 18S and 28S rRNA genes in isolates from 4 fine-grained mutation accumulation lines of *Daphnia obtusa* sampled at 7 time points between generation 5 and 85. Vertical black bars are one standard deviation.
* = difference statistically significant after Bonferroni correction.

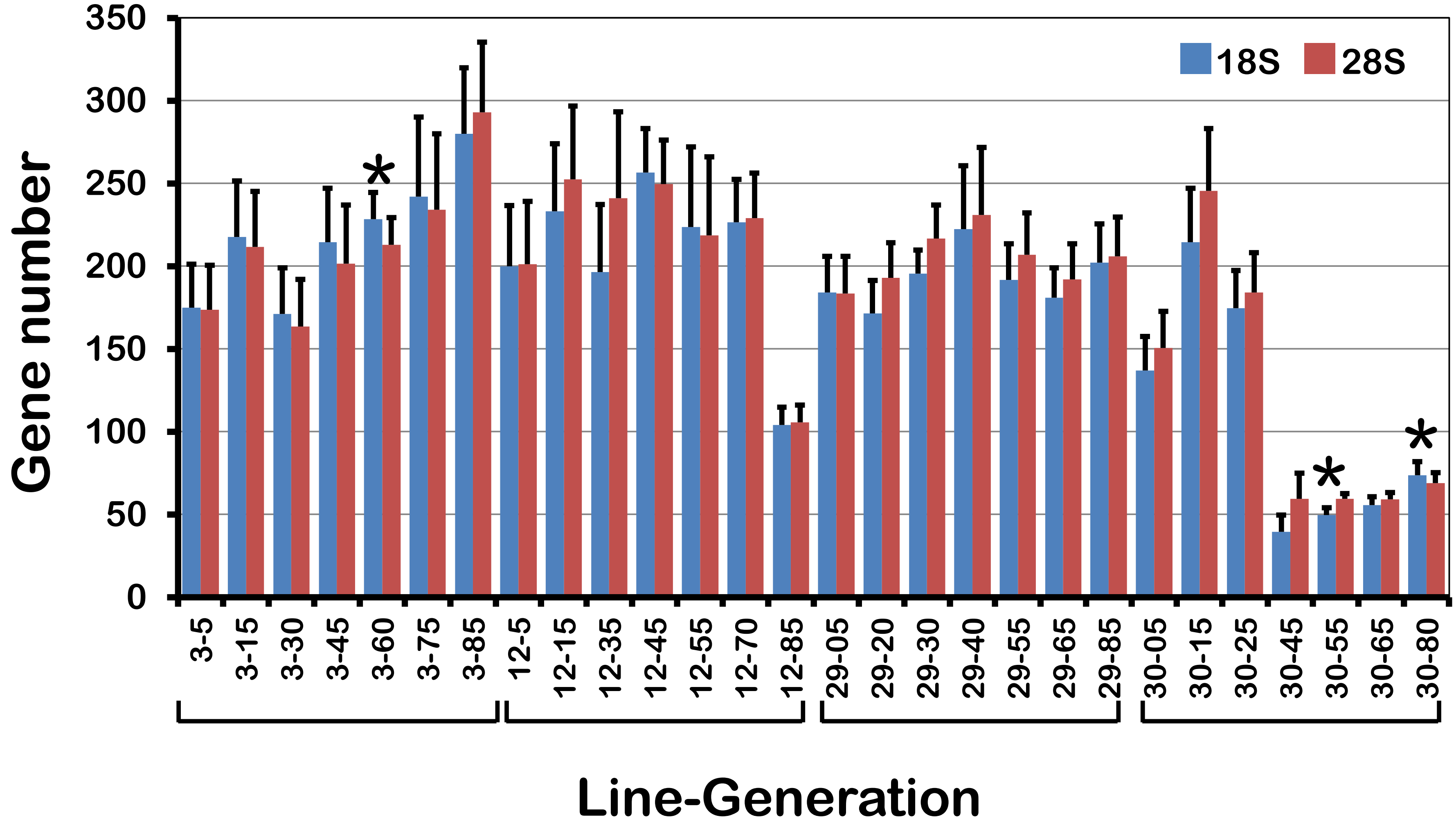

Figure S3. Number of 18S and 28S rRNA genes in isolates from 20 mutation accumulation lines of *Daphnia obtusa* sampled at generation ~87. Vertical black bars are one standard deviation.

* = difference is statistically significant after Bonferroni correction.

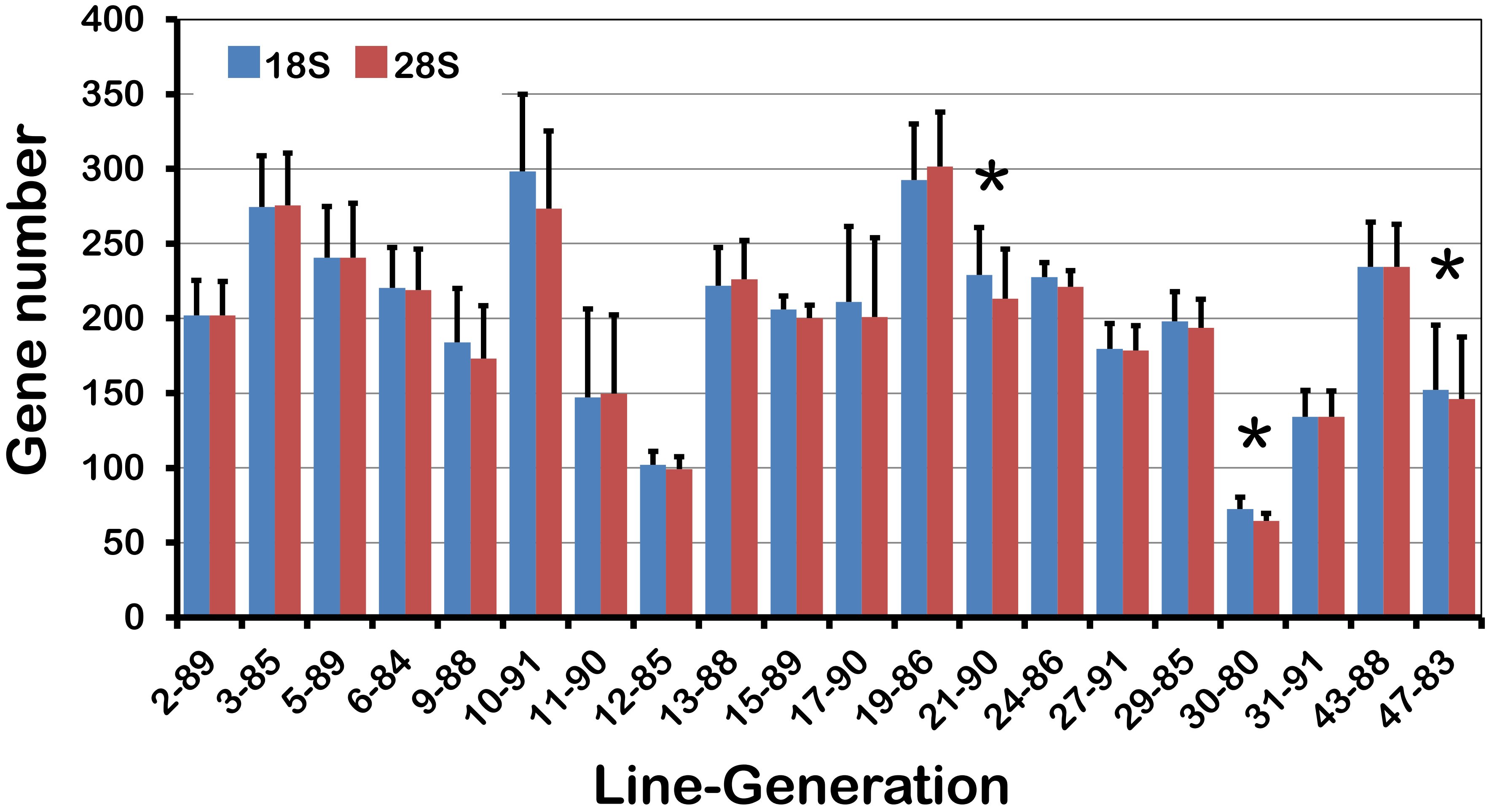

Figure S4. Duplicate estimates of total 28S rRNA number gene (t28S.1, t28S.2) in all *Daphnia obtusa* mutation accumulation line isolates.

MAL-FG = 4 fine-grained mutation accumulation lines each sampled at 7 time points between generation 5 and 85.

MAL-87 = 20 lines sampled at approximately generation 87.

Vertical black bars are one standard deviation.

***** = difference is statistically significant after Bonferroni correction.

Figure S4.

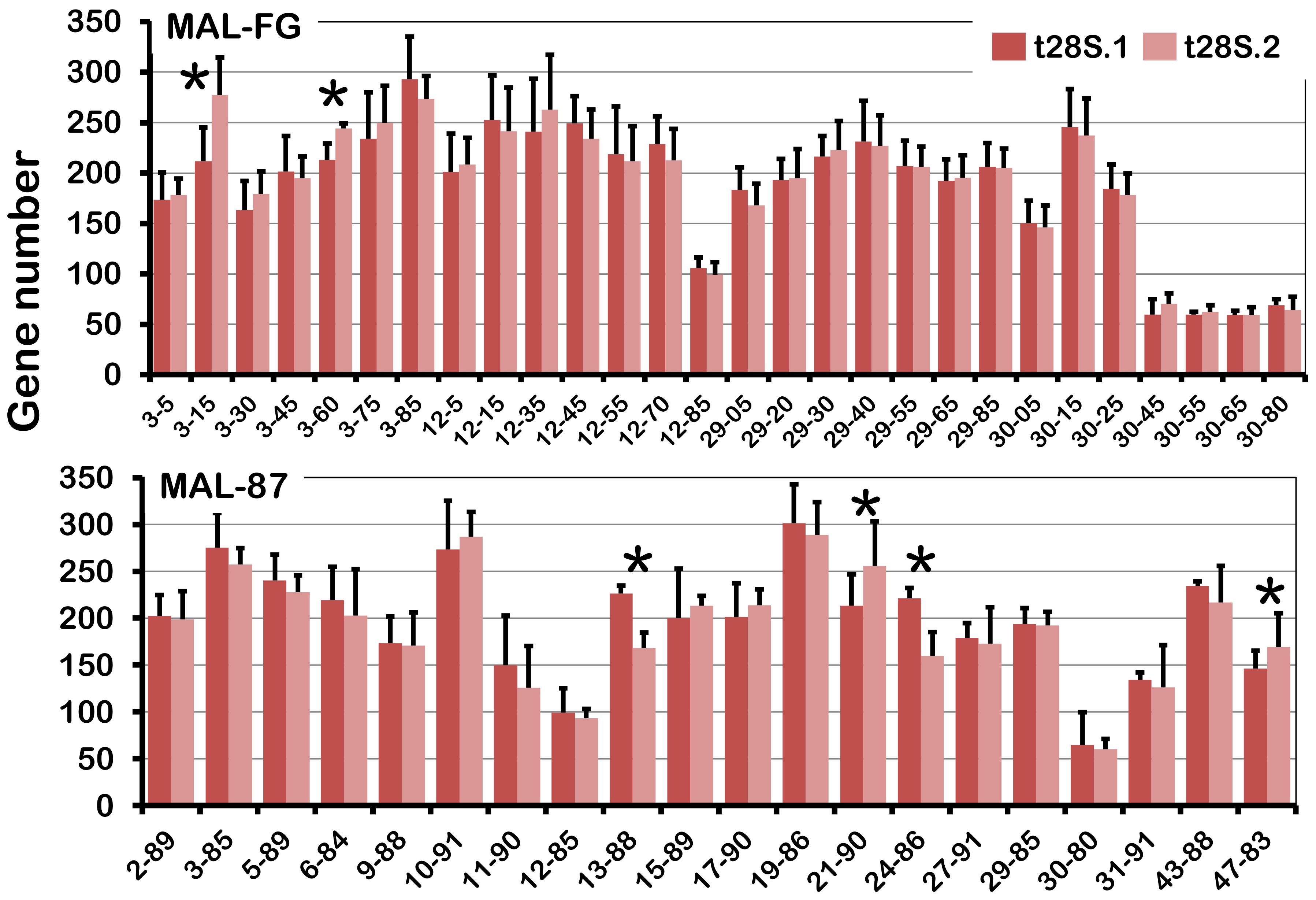
MAL-FG
t28S.1
t28S.2
Gene number
350
300
250
200
150
100
50
0
*
*
3-5
3-15
3-30
3-45
3-60
3-75
3-85
12-5
12-15
12-35
12-45
12-55
12-70
12-85
29-05
29-20
29-30
29-40
29-55
29-65
29-85
30-05
30-15
30-25
30-45
30-55
30-65
30-80
MAL-87
*
*
*
*
2-89
3-85
5-89
6-84
9-88
10-91
11-90
12-85
13-88
15-89
17-90
19-86
21-90
24-86
27-91
29-85
30-80
31-91
43-88
47-83

Figure S5. Regression of duplicate estimates of t28S number (t28S.1, t28S.2) in all *Daphnia obtusa* mutation accumulation line isolates.
$R^2 = 0.87$, $p = 3.0E\text{-}20$, slope = 0.96

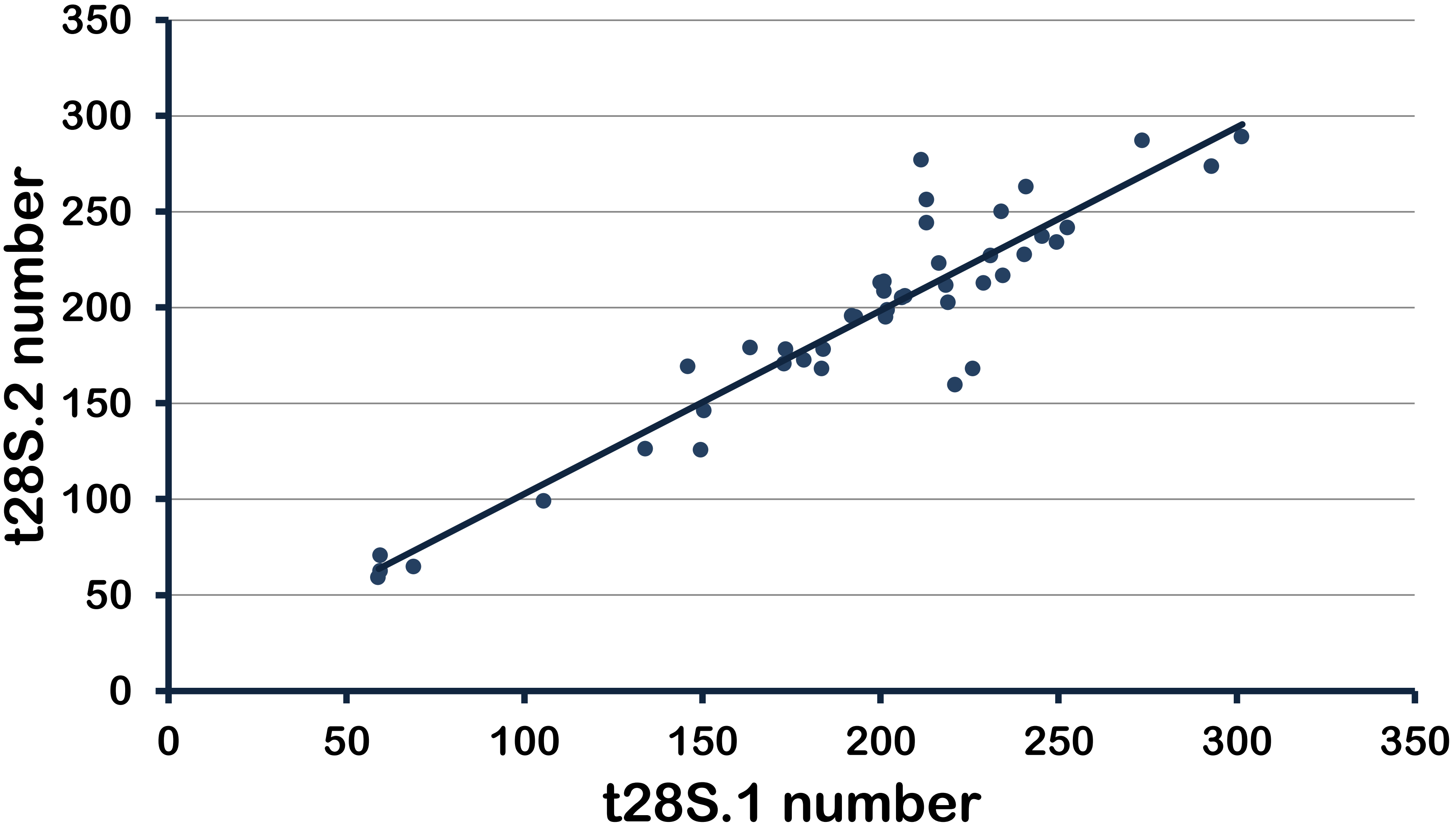

Figure S6. Number of 18S and 28S rRNA genes in *Daphnia obtusa* isolates from natural populations. The four isolates whose 28S number is shown in a lighter shade were all run on the same qPCR plate (see text). Vertical black bars are one standard deviation.
* = difference is statistically significant after Bonferroni correction.

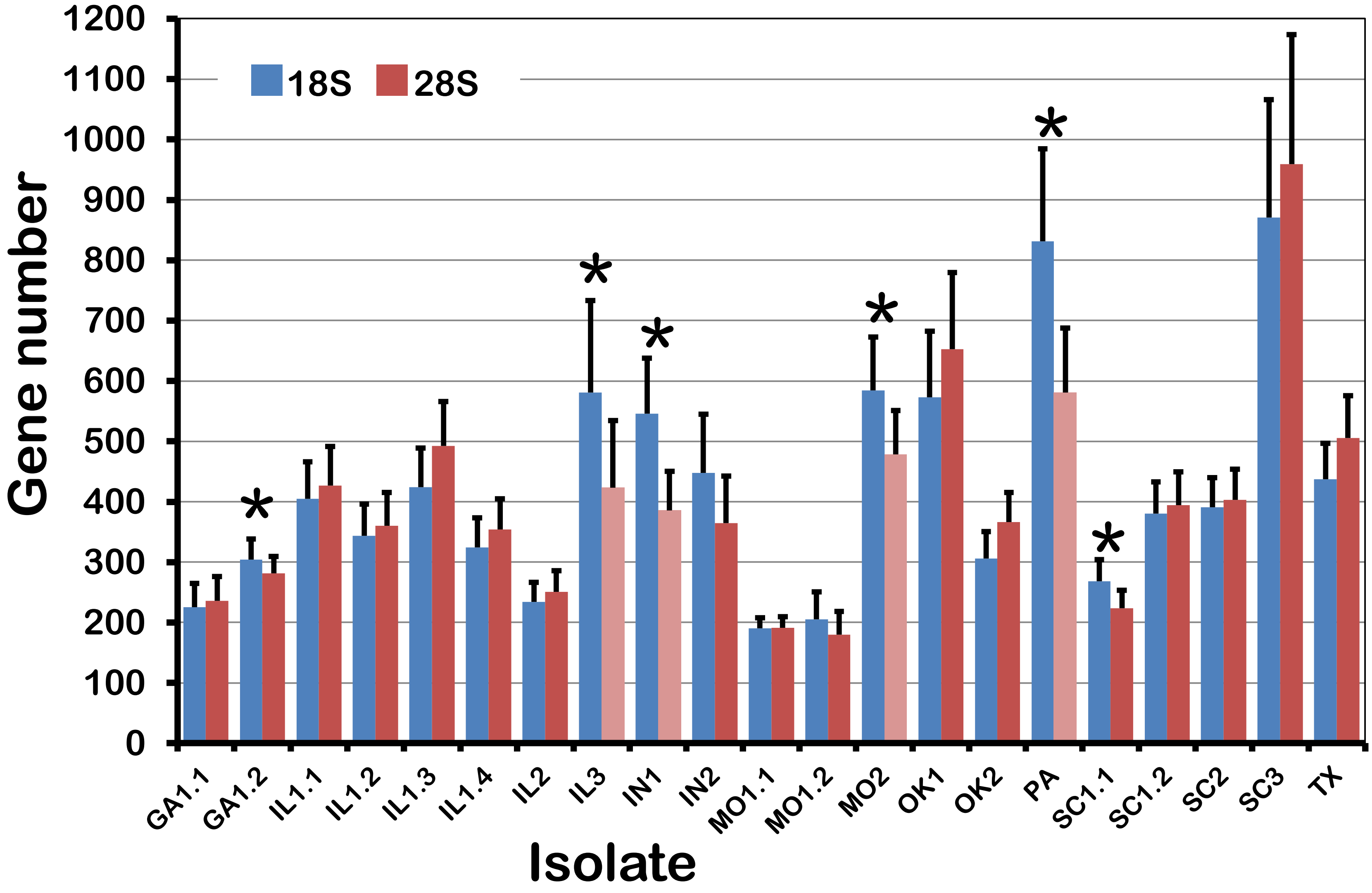

Figure S7. Number of *Pokey* and 28S rRNA genes without inserts (u28S) in *Daphnia obtusa* isolates from 4 fine-grained MAL sampled at 7 time points between generation 5 and 85. rInserts = t28S - (u28S+r*Pokey*A+r*Pokey*B).

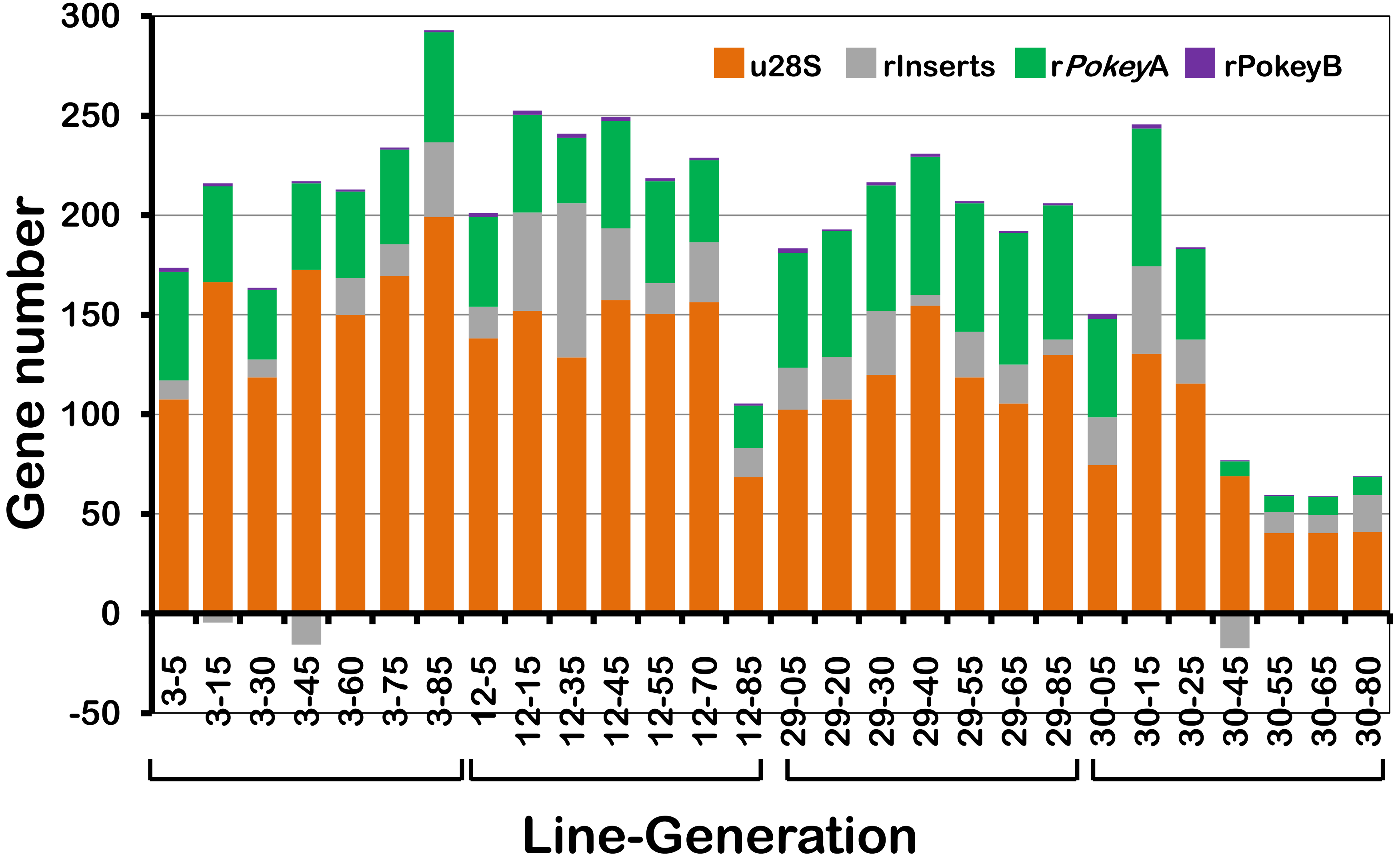

Figure S8. Number of *Pokey* and 28S rRNA genes without inserts (u28S) in *Daphnia obtusa* isolates from 20 mutation accumulation lines sampled at generation ~87. rInserts = t28S - (u28S+r*Pokey*A+r*Pokey*B).

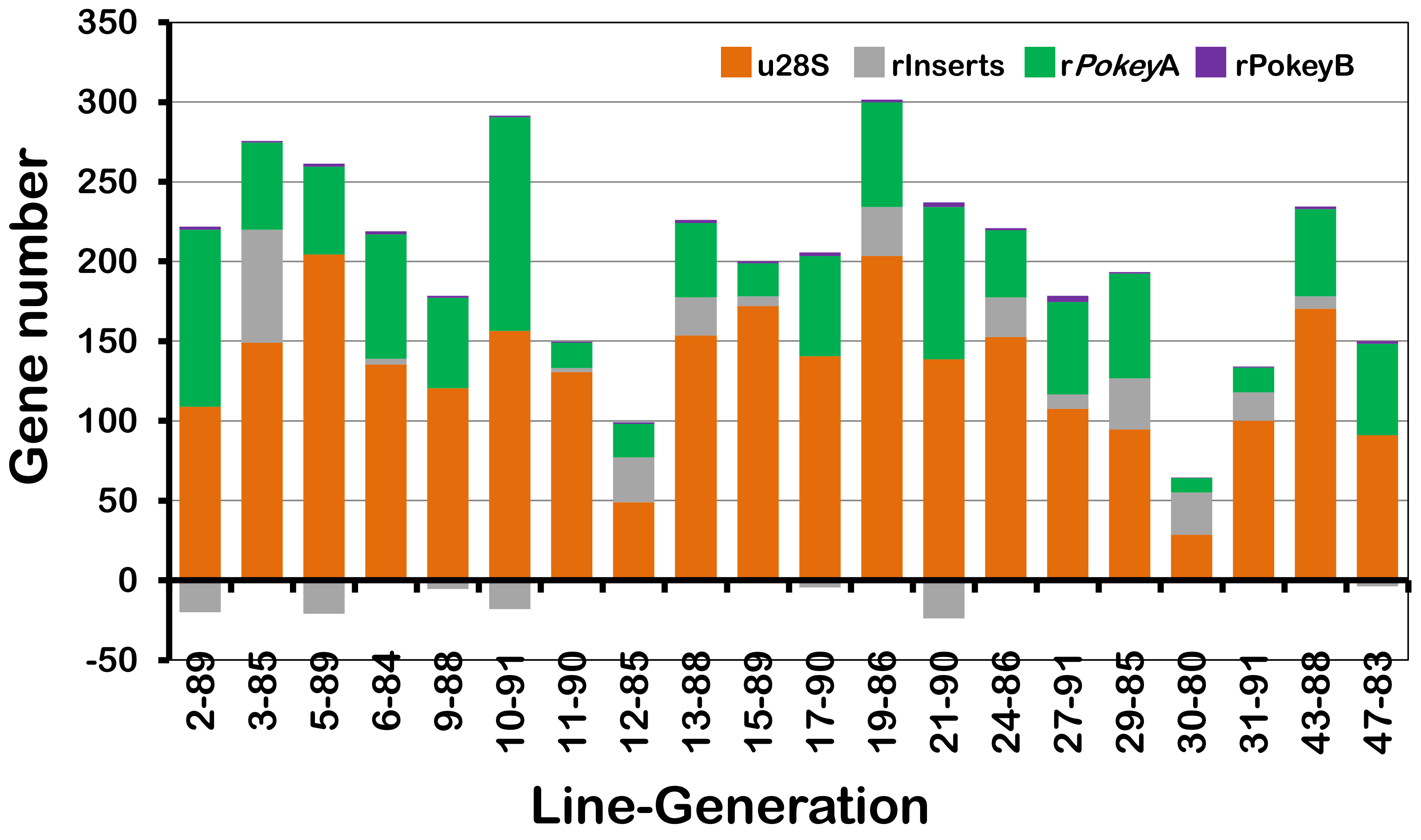

Figure S9. Number of *Pokey* and 28S rRNA genes without inserts (u28S) in *Daphnia obtusa* isolates from natural populations.
rInserts = t28S - (u28S+r*Pokey*A+r*Pokey*B).

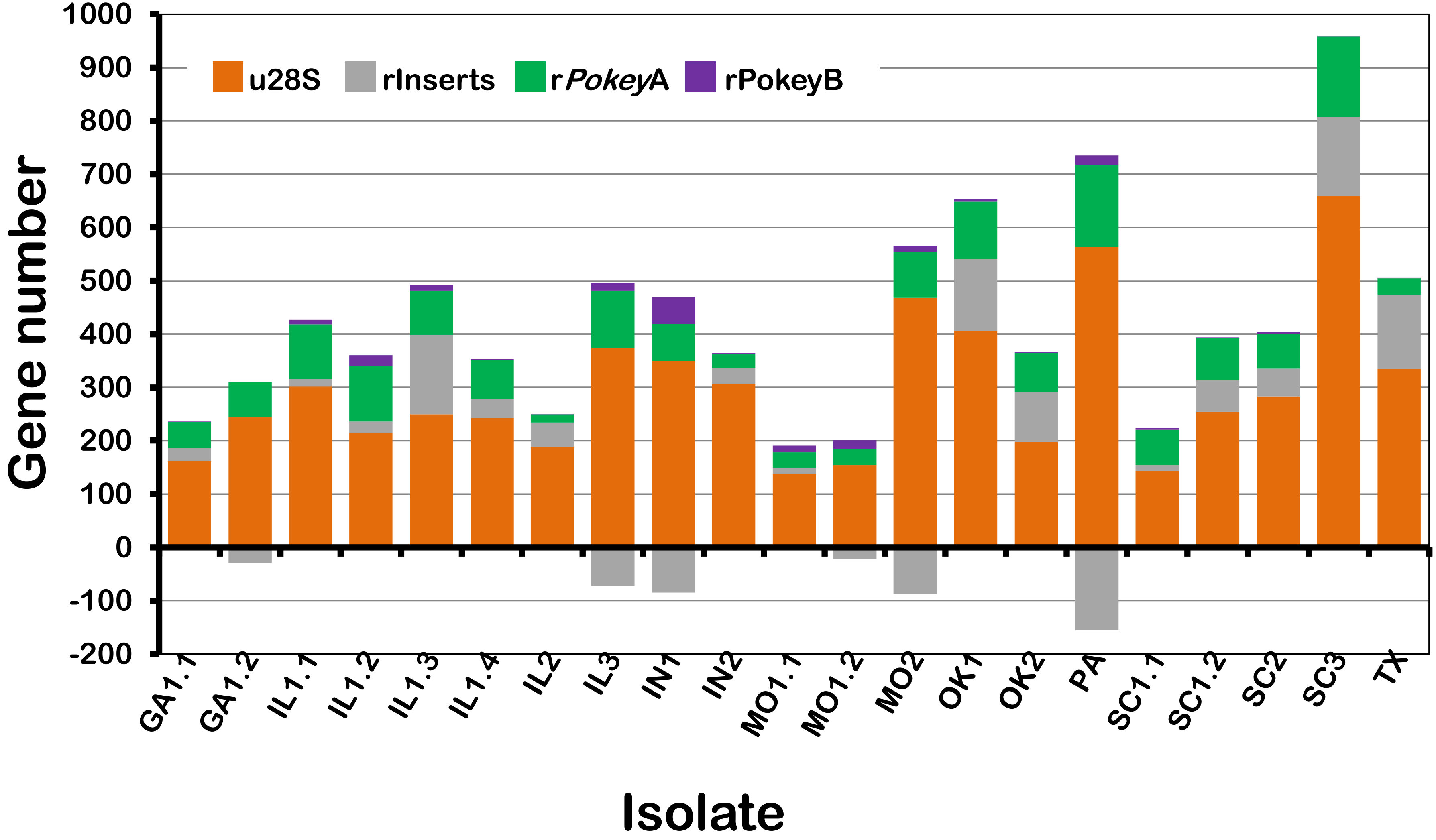

Figure S10.

Regression of rRNA gene and r*Pokey*A number in 4 fine-grained *Daphnia obtusa* mutation accumulation lines sampled at 7 time points between generatioin 5 and 85.

18S = 18S rRNA genes

t28S = total 28S rRNA genes

u28S = 28S rRNA genes lacking Pokey

r*Pokey*A = *Pokey*A inserts in rRNA genes

Figure S10.

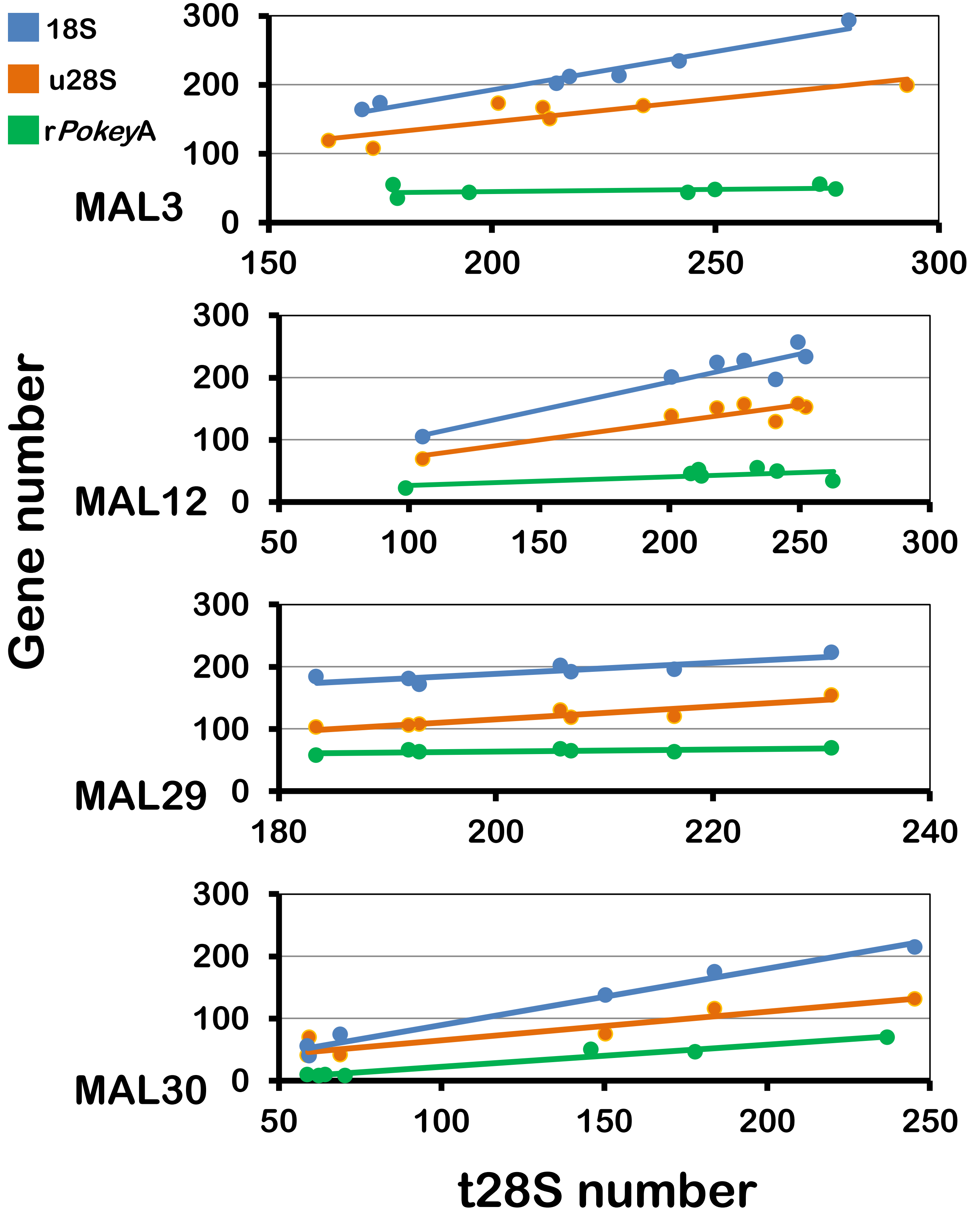

18S
u28S
r*Pokey*A
Gene number
MAL3
300
200
100
0
150
200
250
300
MAL12
300
200
100
0
50
100
150
200
250
300
MAL29
300
200
100
0
180
200
220
240
MAL30
300
200
100
0
50
100
150
200
250
t28S number
